# Childhood cancer incidence by migrant background in Sweden (1991–2021): a nationwide cohort study

**DOI:** 10.1016/j.lanepe.2026.101621

**Published:** 2026-02-20

**Authors:** Genevieve Allen, Elena Extrand, Siddartha Aradhya, Hanna Mogensen, Hannah L. Brooke

**Affiliations:** a Medical Epidemiology, Department of Surgical Sciences, Uppsala University, Uppsala, Sweden; b Stockholm University Demography Unit (SUDA), Department of Sociology, Stockholm University, Stockholm, Sweden; c Department of Immunology, Genetics and Pathology, Cancer Precision Medicine, Uppsala University, Uppsala, Sweden

**Keywords:** Cancer epidemiology, Childhood cancer incidence, Neoplasms, Migration, Parental country of birth, Health disparities

## Abstract

**Background:**

Studies indicate that incidence of certain childhood cancers is lower in low-compared to high-income countries. By examining incidence in migrant populations in Sweden, we aimed to disentangle whether this is due to methodological factors, such as underreporting, or to genetic or environmental aetiology.

**Methods:**

In this nationwide, population-based cohort study, we grouped all children born in Sweden (1990–2019) as: Swedish background, 2nd generation, 2.5 generation (one foreign-born, one Swedish-born parent), or 3rd generation. Age-standardized incidence rates (ASR) for first childhood cancer identified in the National Cancer Register (ages 1–19) were calculated using the 2013 European standard population and are reported per 100,000 person-years [95% confidence intervals]. Incidence rate ratios (IRR) were calculated using Poisson regression adjusted for year of birth and sex. Using the same methods, we examined childhood cancer incidence by parental lineages separately, parental length of stay before birth, and World Bank income classification of parental country of birth.

**Findings:**

During 39,862,393 person-years of follow-up, 6797 children were diagnosed with cancer. The ASR of childhood cancer in Swedish background children was 17.18 [16.63–17.74], this was slightly higher in boys (18.20 [17.42–19.01]) than girls (16.09 [15.34–16.88]). The overall results were largely similar across migrant generations and parental length of stay. Lymphoma incidence was higher in 2nd generation (ASR 3.09 [2.53–3.78]) compared to Swedish background children (2.22 [2.02–2.44]). Children with migrant mothers from high-income countries had a higher incidence of leukaemia (ASR 5.88 [4.78–7.24]) compared to Swedish background children (4.31 [4.09–4.55]), whereas central nervous system tumour and leukaemia incidence was lower in children with mothers from low-income countries (2.64 [1.79–3.88], 3.66 [2.76–4.85], respectively) compared to Swedish background children (4.49 [4.24–4.75], (4.31 [4.09–4.55]). The pattern was similar for fathers from low-income countries and for IRRs.

**Interpretation:**

Finding lower incidence of CNS tumours and leukaemia among children born in Sweden to mothers from low-income countries challenges the hypothesis that low incidence of some childhood cancers in low-income countries is solely due to underreporting, but rather suggest that genetic or environmental factors may underlie these observations.

**Funding:**

Barncancerfonden (PR2023-0060).

**Research in context**

**Evidence before this study**

We searched PubMed without language restrictions for studies on incidence of childhood cancer in migrant populations from 2000 to 2026 for the following MeSH terms in the title or abstract: “Neoplasms”, “Cancer”, “Malignancy” AND “Child”, “Children”, “Pediatric” AND “Emigrants and Immigrants”, “Immigrant”, “Migration”, “Refugee” AND “Incidence”, “Epidemiology”, “Risk”, “Prevalence”. We also searched for global trends in childhood cancer incidence using the search ((“Neoplasms” [MeSH]) AND (“Child” [MeSH] OR “Pediatrics” [MeSH]) AND (“Incidence” [MeSH]) AND (“Global Health” [MeSH] OR global [tiab] OR international [tiab] OR “Developing Countries” [MeSH] OR LMIC [tiab] OR worldwide [tiab])) AND ((“2000/01/01” [Date–Publication]: “2026/02/02” [Date–Publication])).

The evidence describing childhood cancer incidence in migrant populations is limited. Studies in the United States focus on Hispanic populations and have shown variation in incidence related to mothers’ country of birth. In Germany, one study found similar incidence of childhood cancer in children with a Turkish background compared to children with a German background.

A Swedish study including children diagnosed from 1961 to 1998 found similar incidence of most childhood cancers in children of immigrants compared to children of Swedes. They identified a higher rate of non-Hodgkin's lymphoma in children of parents from Turkey and Yugoslavia. However, in the years since this publication, Sweden has under gone a large demographic transition with an influx of migrants from the Middle East in the 2010s. Additionally, the previous article did not differentiate between children with one foreign-born parent and one Swedish-born, or by parental length of stay in Sweden prior to the child's birth. No study in the Nordic context has investigated the incidence of childhood cancer among the third generation children.

Globally, studies have shown lower rates of childhood cancer in countries with a low human development index and low World Bank income level. It is unknown whether this is due to methodological factors, such as limited diagnostic capabilities or incomplete cancer registration, or whether it reflects aspects of childhood cancer aetiology, such as genetic or environmental determinants.

**Added value of this study**

The present study uses high-quality, nationwide register data to link Swedish-born children to their parents and grandparents allowing us to study three generations and nuances in the diverse population of children with a migrant background. Additionally, children in Sweden have universal access to healthcare and registration of cancer is essentially complete meaning any difference in incidence is unlikely due to incomplete registration or due to underdiagnosis.

**Implications of all the available evidence**

Our findings in the Swedish context do not support the hypothesis that lower incidence of CNS tumours and leukaemias in low-income countries is solely due to underreporting, but rather suggest that genetic or environmental factors may underlie these observations. This highlights the need for future research on childhood cancer aetiology to include data from low-income countries.

## Introduction

Global studies of childhood cancer incidence found that the two most prevalent cancer diagnoses in children, leukaemias and central nervous system (CNS) tumours, have a lower incidence in low-income countries compared to high-income countries.^1^, ^2^, ^3^, ^4^ These trends could represent true differences, offering insight into the genetic or environmental aetiology of childhood cancer. They could also represent a combination of limited diagnostic capabilities and incomplete cancer registration in low-income countries, which may be contributed to by higher infant mortality rates, limited access to healthcare facilities, mis-diagnosis or under-recognition of cancer symptoms, reliance on traditional medicine, as well as less developed data infrastructures and registration procedures.

In Sweden there is mandatory reporting of incident cancers in a nationwide cancer register and state-of-the-art diagnostic capabilities. Studying cancer incidence among children with a migrant background in this context will shed light on the extent to which incomplete reporting and limited diagnostic capabilities could be distorting true incidence of childhood cancer in low-income countries. One previous study in this context that is now over 20 years old found a higher incidence of lymphoma in Swedish children with parents from the former Yugoslavia and Turkey compared to children with parents born in Sweden.^5^ This, however, was based on a dozen cases and did not explore the heterogeneity of migrant families, such as income level of the parental country of origin and parental length of stay in Sweden; nor did they examine the grandchildren of migrants (third generation). In the interim, Sweden has experienced an influx of migrants from the Middle East and Northern Africa (MENA). The proportion of children under 18 years of age in Sweden with at least one foreign born parent in 2023 was 33%.^6^^,^^7^

Studies in migrant populations also provide clues to disease aetiology. For example, the aetiology of childhood asthma and type 1 diabetes is thought to have an environmental component, since the incidence of these disease differs depending on how long migrant parents reside in a host country prior to the child's birth.^8^^,^^9^ These studies demonstrated that for parents from countries with a low incidence of asthma and type 1 diabetes but a long length of residence in a host country with a higher incidence, the incidence rate in their children rose with parental length of stay.^8^^,^^9^ This suggests that longer exposure to the host country environment subsequently affects the health of children. In childhood cancer, one study found that children of non-US born Hispanic mothers retained the cancer risk of the mother's country of birth.^10^ It is unknown, however, if parental length of residence in a host country affects the risk for childhood cancer in offspring.

To disentangle if the differences in childhood cancer incidence between low- and high-income countries are due to underreporting or related to aetiology, we leveraged the mandatory and essentially complete registration of cancer diagnoses in Sweden to describe the incidence of childhood cancer in children born in Sweden by migrant background between 1991 and 2021.

## Methods

### Study population

**Fig. 1:**
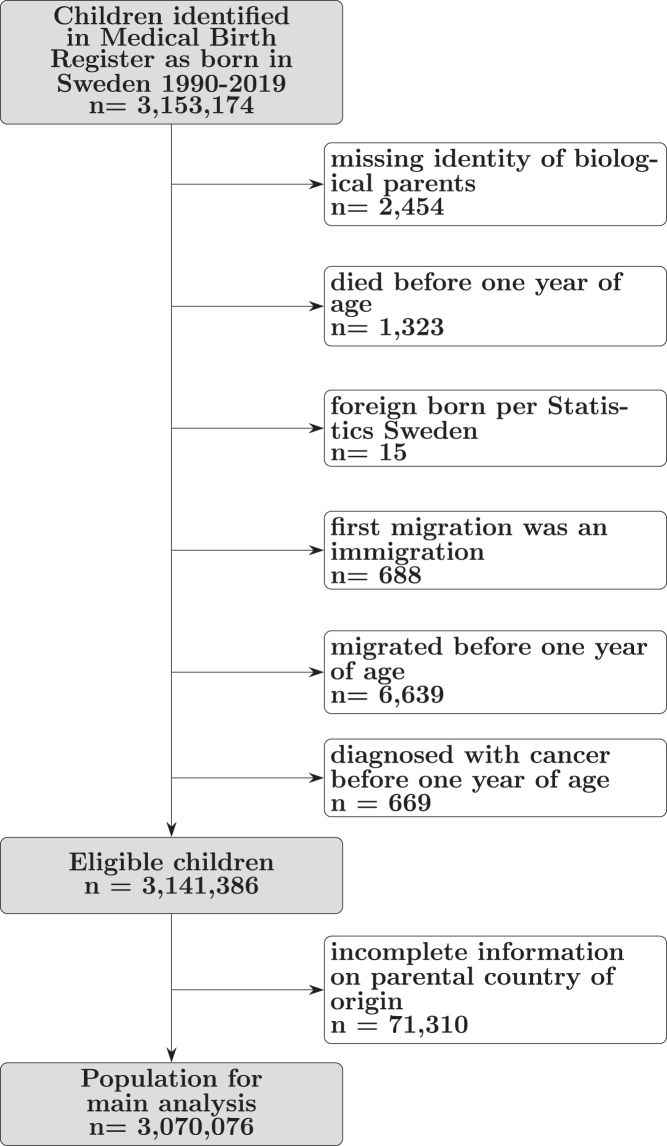
**Selection of study population**.

This nationwide population-based cohort study included all children born in Sweden during 1990–2019 who were alive and registered in Sweden at the end of their first calendar year, with no previous cancer diagnosis (n = 3,141,386), identified from the National Medical Birth Register. We excluded children without any identifiable biological parents (n = 2454), those who died before one year of age (n = 1323), those with errors in registration (n = 703), those who migrated before one year of age (n = 6639), and those diagnosed with cancer before one year of age (n = 669) (Fig. 1). Children were linked to their parents and grandparents in the Multigeneration Register.^11^ Linkages were made possible via the unique personal identity number assigned to all residents planning to reside in Sweden at least one year.

The Multigeneration Register includes index persons born 1932 onward and alive in 1961.^11^ For individuals born in Sweden, information on biological mother is available for 97% and father for 95% of index persons.^11^ There was incomplete information on parental country of origin for 71,310 children.

### Exposure assessment

Children were considered of Swedish background if they had two Swedish-born parents and four Swedish-born grandparents; third generation if they had two Swedish-born parents and at least one foreign-born grandparent; 2.5 generation if they had one foreign-born and one Swedish-born parent; and second generation if they had two foreign-born parents. These categories have been used in previous literature.^12^^,^^13^ Children were excluded from the main analysis if they were missing information on parental country of origin (n = 71,310) (Fig. 1).

To incorporate the heterogeneity of migrant populations, we considered secondary exposures by maternal and paternal lineages separately for: migrant background, parental length of stay prior to the child's birth, and the World Bank income classification of the parental country of birth.

For the maternal lineage analysis, children were considered Swedish background if their mother and maternal grandparents were born in Sweden, third generation if their mother was born in Sweden and at least one maternal grandparent was born abroad, and second generation if their mother was foreign-born. The same process was completed for paternal lineage.

For second generation children in the maternal lineage analysis, maternal length of stay was calculated as the time, in years, from first maternal immigration to Sweden to the child's birth. This was then categorised by five-year time increments (less than five years, 5–9 years, 10–14 years, 15+ years). Five-year increments were chosen to align with relevant migration phases, i.e., recent arrivals, early integration period, mid-term residents, and long-term residents, while also ensuring a sufficient number of cases in each category. There was no change to Swedish background children. Second generation children were excluded if data were missing on maternal immigration date or there were errors in maternal registration (Supplementary Figure S1). The same process was completed for second generation children by paternal lineage (Supplementary Figure S1).

World Bank income level of the parental country of birth was based on gross national income per capita (Atlas method)^14^ from the year of immigration to Sweden for mothers and fathers separately. Countries were classified as low-, middle-, and high-income. See supplementary methods for details.

### Outcome assessment

Beginning at the child's first birthday, outcome data were obtained from the National Cancer Register, a nationwide register with obligatory and complete nationwide reporting of cancer since 1958.^15^

We identified all first malignant cancers, as well as non-malignant CNS tumours (malignant by site)^16^ diagnosed in our study population between the ages of 1–19 in the years 1991–2021. Cancers were categorised by morphology and topography according to the International Classification of Childhood Cancer (ICCC3) nomenclature.^17^

### Covariates

A directed acyclic graph was drawn to identify the assumed causal framework relating migrant background to incidence of childhood cancer (Supplementary Figure S2). We adjusted for year of birth (extracted from the Medical Birth Register and treated as a continuous variable) and sex (assigned at birth) in the Poisson models, as we identified these as outcome predictors. However, potential mediators, such as maternal and paternal age and occupation, were not adjusted for, enabling us to estimate the total effect of migrant background on incidence of childhood cancer.

### Statistical analysis

Participants were followed from their first birthday until cancer diagnosis, migration, death, 20th birthday, or end of study period (December 31, 2021). Immigration and emigration events were obtained from the Total Population Register which captures 95% of immigrations and 91% of emigrations within 30 days.^18^ The date of death was obtained from the National Cause of Death register, which dates back to 1952.^19^

Age-standardized incidence rates (ASR) reported per 100,000 person-years were calculated using the direct method from age-specific rates (1–4 year-olds, 5–9 year-olds, 10–14 year-olds, and 15–19 year-olds) and the 2013 European standard population^20^ for the overall incidence of childhood cancer and major ICCC3 subtypes (I. Leukaemias, II. Lymphomas, III. Central Nervous System (CNS)). Due to low incidence, groups IV-XI were grouped together as Other Solid Tumours.

The confidence intervals (CI) were based on the normal approximation of the logarithm of the rate with the variance of the log rate derived using the delta method. Both confidence intervals and ASR were calculated using the PopEpi package in R, which was the only algorithm used for this.^21^

To describe relative incidence across migrant background, incidence rate ratios (IRR) with 95% confidence intervals were calculated for childhood cancer and major ICCC3 subtypes using Poisson regression. We used generalised linear models with a Poisson distribution and log link. Incident cancer was a count variable (0, 1) and log person time was included as an offset in order to calculate events per unit of time. This approach assumes that events follow a Poisson distribution, that the mean rate is a log linear function of the covariates, and that event counts are conditionally independent given the covariates. All models were adjusted for sex and year of birth.

ASR and IRR for incident childhood cancer and major ICCC3 subtypes for the secondary exposures of maternal/paternal background separately, length of stay, and World Bank income level (low-, middle- and high-income) of parental country of origin were calculated as described above.

### Sensitivity analysis

Due to known differences in childhood cancer incidence by sex, we repeated all analysis described above for males and females separately. In sex-specific analysis, IRR models were only adjusted for year of birth.

Due to prior reports of increased incidence of leukaemia, lymphoma, and CNS tumour ICCC3 subtypes, such as acute lymphoblastic leukaemia (ALL) and Hodgkin's lymphoma, in certain subpopulations,^1^^,^^3^^,^^5^ we calculated ASR and IRR for leukaemia (acute lymphoid leukaemia, acute myeloid leukaemia, chronic myeloid leukaemia/myelodysplastic syndromes/unspecified leukaemia), lymphoma (Hodgkin lymphoma, Non-Hodgkin lymphoma, Burkitt/miscellaneous lymphomas), and CNS (ependymomas, astrocytomas/other gliomas, intracranial and intraspinal embryonal tumours, other/unspecified CNS tumours) subtypes by migrant background, by World Health Organization region of parental country of birth, and by World Bank income level (low-, middle-, and high-income) of parental country of birth.

All analyses were run on R version 4.2.1.^22^

### Ethical approval

The Regional Ethical Review Board, Uppsala, Sweden, granted ethical approval for the study (Dnr 2018/274, approval date: 2018.08.15, with amendments, specifically: Dnr 2024-00926-02, with approval date: 2024-02-27). The requirement for informed consent was waived for this study since it was based on de-identified data from national registers. This study was performed in accordance with the Declaration of Helsinki. Data were used with permission from the register holders at Statistics Sweden and the National Board of Health and Welfare (Socialstyrelsen). The study is reported in accordance with the Strobe guidelines.

### Role of the funding source

The funders had no role in study design; data collection, analysis, or interpretation; writing of the manuscript; nor in the decision to submit the manuscript for publication.

## Results

### Main analysis

The main analysis included 3,070,076 children (Fig. 1) representing 39,862,393 person-years of observation. Median follow up time was 14.68 years (IQR 7.42–19.00) (Supplementary Table S1). Fifty-six percent (n = 1,727,661) of children were Swedish background (Table 1). Fifteen percent (n = 457,316) were second-generation children, of which about half were born 2010–2019 (Table 1). Middle East and North African countries were the most common parental region of origin among the second-generation. Thirteen percent (n = 386,330) and 16% (n = 498,769) were 2.5 and third-generation children, respectively.

**Table 1:** Characteristics of children born in Sweden 1990–2019 by migrant background.

Migrant background	Swedish background, N = 1,727,661	3rd generation, N = 498,769	2.5 generation, N = 386,330	2nd generation, N = 457,316	Overall, N = 3,070,076
**Sex**^a^					
Male	888,329 (51%)	256,543 (51%)	198,334 (51%)	234,868 (51%)	1,578,074 (51%)
Female	839,332 (49%)	242,226 (49%)	187,996 (49%)	222,448 (49%)	1,492,002 (49%)
**Birth cohort**					
1990–1999	616,818 (36%)	169,274 (34%)	112,739 (29%)	108,290 (24%)	1,007,121 (33%)
2000–2009	555,703 (32%)	160,664 (32%)	122,272 (32%)	128,638 (28%)	967,277 (32%)
2010–2019	555,140 (32%)	168,831 (34%)	151,319 (39%)	220,388 (48%)	1,095,678 (36%)
**Father's Region of Birth**					
Sweden	1,727,661 (100%)	498,769 (100%)	180,882 (47%)	0 (0%)	2,407,312 (78%)
Nordic, except Sweden	0 (0%)	0 (0%)	43,357 (11%)	19,117 (4.2%)	62,474 (2.0%)
Non-Nordic European	0 (0%)	0 (0%)	73,820 (19%)	137,748 (30%)	211,568 (6.9%)
Middle East and North Africa	0 (0%)	0 (0%)	30,638 (7.9%)	163,815 (36%)	194,453 (6.3%)
Sub-Saharan Africa	0 (0%)	0 (0%)	12,062 (3.1%)	70,197 (15%)	82,259 (2.7%)
Asia	0 (0%)	0 (0%)	12,919 (3.3%)	47,312 (10%)	60,231 (2.0%)
Americas	0 (0%)	0 (0%)	29,409 (7.6%)	18,638 (4.1%)	48,047 (1.6%)
Oceania	0 (0%)	0 (0%)	3243 (0.8%)	489 (0.1%)	3732 (0.1%)
**Mother's Region of Birth**					
Sweden	1,727,661 (100%)	498,769 (100%)	205,448 (53%)	0 (0%)	2,431,878 (79%)
Nordic, except Sweden	0 (0%)	0 (0%)	44,082 (11%)	20,471 (4.5%)	64,553 (2.1%)
Non-Nordic European	0 (0%)	0 (0%)	52,735 (14%)	141,839 (31%)	194,574 (6.3%)
Middle East and North Africa	0 (0%)	0 (0%)	9943 (2.6%)	156,212 (34%)	166,155 (5.4%)
Sub-Saharan Africa	0 (0%)	0 (0%)	6531 (1.7%)	68,188 (15%)	74,719 (2.4%)
Asia	0 (0%)	0 (0%)	43,113 (11%)	51,386 (11%)	94,499 (3.1%)
Americas	0 (0%)	0 (0%)	23,304 (6.0%)	18,924 (4.1%)	42,228 (1.4%)
Oceania	0 (0%)	0 (0%)	1174 (0.3%)	296 (0.1%)	1470 (0.05%)

a Variable distributions are expressed as n (%).

**Fig. 2:**
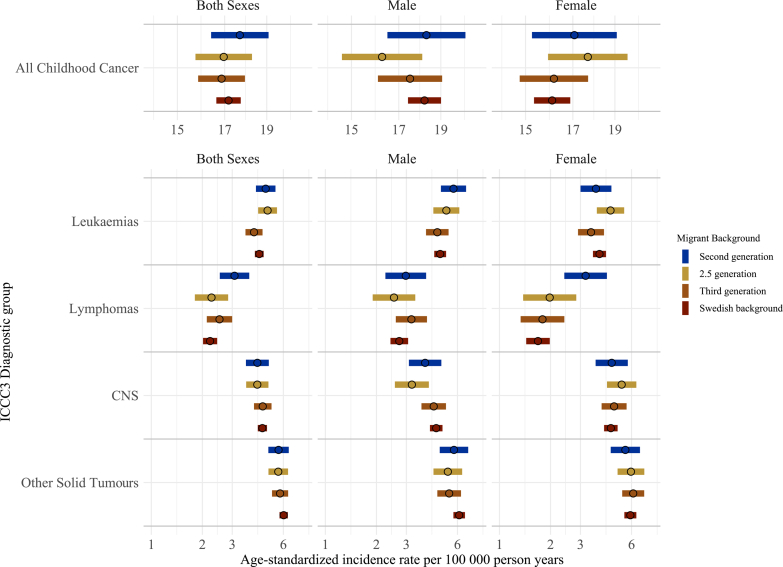
**Age-standardised incidence rates for ICCC3 childhood cancer diagnostic groups in Sweden 1991–2021 among children aged 1–19 years by migrant background, both sexes combined and stratified by child's sex.** CNS, central nervous system; ICCC3, International Classification of Childhood Cancer 3. Age standardisation was calculated via direct standardisation using the 2013 European standard population.

Between 1991 and 2021, 6797 children in our population were diagnosed with a childhood cancer at a median age of 7.5 years (interquartile range 3.4 years to 14.4 years). This represents an overall crude incidence rate of 17.05 cases per 100,000 person-years. Overall, incidence rates were higher for males than females (Fig. 2, Supplementary Table S2). The ASR ([95% confidence intervals], reported per 100,000 person-years) of childhood cancer among children with Swedish background was 17.18 [16.63–17.74]. ASR were similar for 3rd, 2.5 and 2nd generation children (Fig. 2). A similar pattern was seen for other solid tumours. No difference across generations was seen in the IRRs for childhood cancer overall and other solid tumours (Supplementary Table S2).

ASR for leukaemia and lymphomas were slightly higher in the second generation compared to Swedish background children. For leukaemia, the rate was 0.39 per 100,000 person-years higher in second generation compared to Swedish background, which represents a 6% higher incidence rate (Fig. 2 and Supplementary Table S2). ASR for lymphoma was 0.87 per 100,000 person-years higher in second generation children (3.09 [2.53–3.78]) compared to Swedish background children (2.22 [2.02–2.44]) and was also reflected in the IRR (1.26 [1.02–1.55). This was more pronounced in females compared to males (Fig. 2) (Supplementary Table S2).

ASR for CNS tumours was slightly lower in the second (4.22 [3.61–4.92]) and 2.5 generation (4.21 [3.62–4.90]) compared to Swedish background children (4.51 [4.24–4.80]), largely driven by the males (Fig. 2), equating to a 10% lower rate (Supplementary Table S2).

### Parental lineage

When migrant background was defined by maternal and paternal lineage separately, 3,121,433 and 3,079,047 children were included in the analysis, respectively (Supplementary Figure S1 and Supplementary Table S3).

**Fig. 3:**
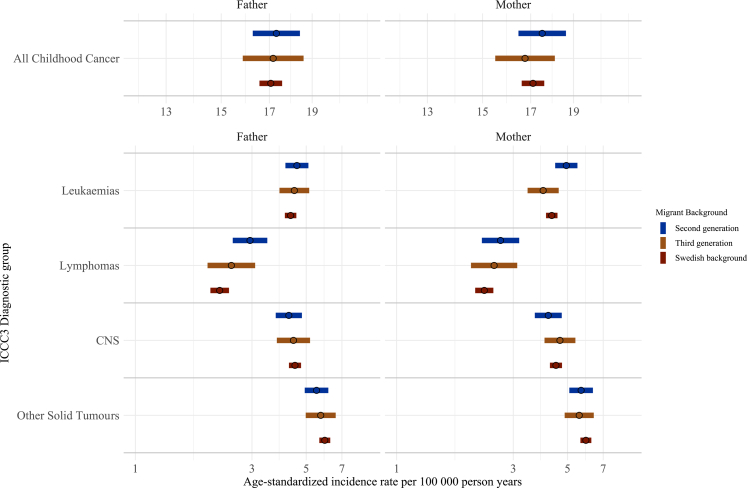
**Age-standardised incidence rates for ICCC3 childhood cancer diagnostic groups in Sweden 1991–2021 among children aged 1–19 years by parental migrant background, stratified by maternal and paternal lineage.** CNS, central nervous system; ICCC3, International Classification of Childhood Cancer 3. Age standardisation was calculated via direct standardisation using the 2013 European standard population.

Results were similar for maternal and paternal lineages and generally followed the pattern seen in the main analysis with slightly higher rates of lymphoma and leukaemia and slightly lower rates of CNS tumours in the second generation compared to Swedish background children (Fig. 3 and Supplementary Table S3).

### Parental length of stay

For children of migrant mothers, the median maternal length of stay in Sweden prior to the child's birth was 6 years (interquartile range 2–14 years) whereas for children of migrant fathers, the median paternal length of stay was 9 years (interquartile range 4–17 years) before the child's birth (Supplementary Table S4).

**Fig. 4:**
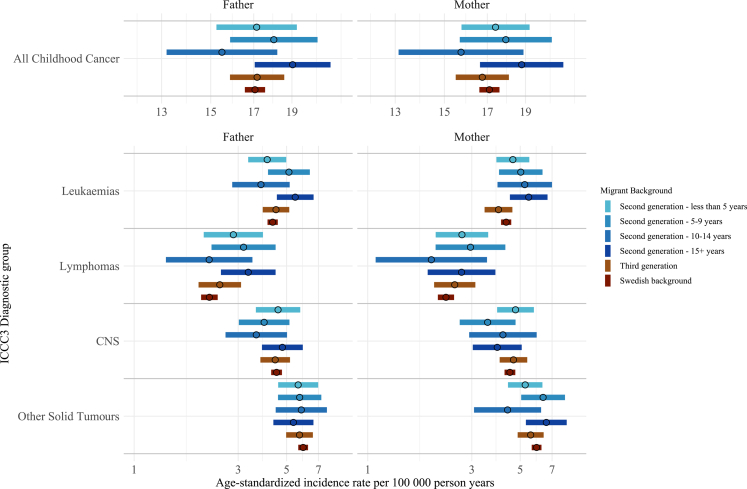
**Age-standardised incidence rates for ICCC3 childhood cancer diagnostic groups in Sweden 1991–2021 among children aged 1–19 years by parental length of stay prior to child's birth.** CNS, central nervous system; ICCC3, International Classification of Childhood Cancer 3. Age standardisation was calculated via direct standardisation using the 2013 European standard population.

There was no clear stepwise pattern for ASR or IRR for childhood cancer by parental length of stay (Fig. 4). Across diagnostic groups, ASRs and IRRs fluctuated without consistent trends in relation to either maternal or paternal duration of residence (Supplementary Table S4).

### World Bank income level of parental country of birth

**Fig. 5:**
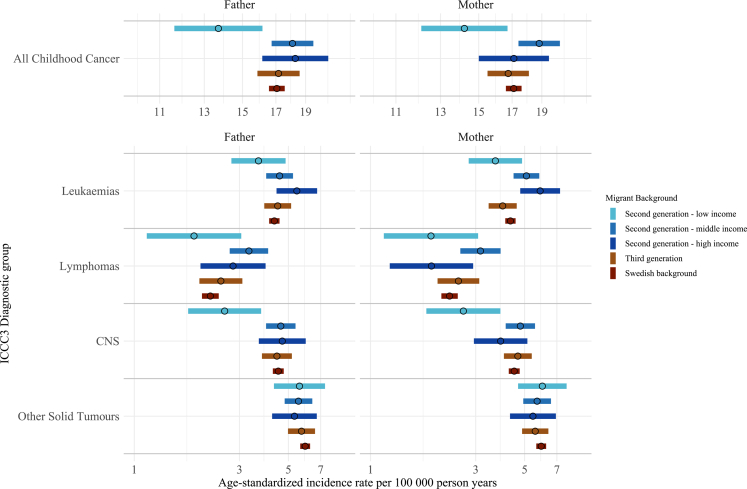
**Age-standardised incidence rates for ICCC3 childhood cancer diagnostic groups in Sweden 1991–2021 among children aged 1–19 years by World Bank income level of parental country of origin.** CNS, central nervous system; ICCC3, International Classification of Childhood Cancer 3. Age standardisation was calculated via direct standardisation using the 2013 European standard population.

The ASR for childhood cancer overall, leukaemias, and CNS tumours for children with a mother born in a low-income country ranged from 0.63 to 2.88 cases per 100,000 person-years lower than the ASR for Swedish background children (Fig. 5).

Children with a mother born in a middle- or high-income country generally had similar ASR to Swedish background children with the exception of leukaemia. For children with a mother born in a middle-income or high-income country, the ASR for leukaemia was 1.10–1.57 cases higher per 100,000 person-years compared to Swedish background children (Fig. 5).

**Fig. 6:**
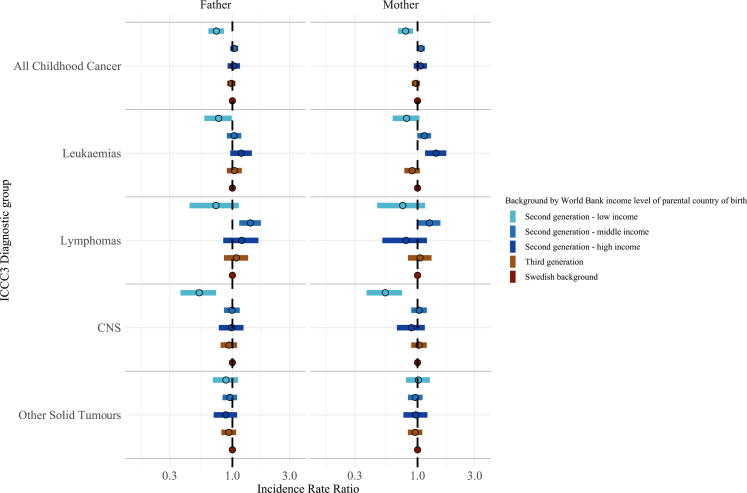
**Incidence rate ratios for ICCC3 childhood cancer diagnostic groups in Sweden 1991–2021 among children aged 1–19 years by World Bank income level of parental country of origin.** IRR, Incidence rate ratios; CNS, central nervous system; ICCC3, International Classification of Childhood Cancer 3. IRR were estimated from multivariable Poisson regression adjusted for sex and year of birth.

The corresponding IRR ranged from 0.54 (0.38–0.74) for CNS tumours in children of mothers from low-income countries compared to children with a Swedish background to 1.42 (1.16–1.74) for leukaemias in children of mothers born in high-income countries (Fig. 6). Results were similar when examined by paternal country of birth (Figs. 5 and 6 and Supplementary Table S5).

### Leukaemia, lymphoma and CNS subtype results

When leukaemia subtypes were examined in detail, ALL represented 1641 of the 2071 leukaemia cases. When examined by migrant background, ASR for ALL was highest in the 2.5 generation (3.66 versus 3.30 for Swedish background children, IRR 1.10 [0.95–1.28]) (Supplementary Table S6). When broken down by WHO region of parental country of birth, ALL incidence was lowest in 2nd generation children with fathers from the WHO African Region (ASR 2.32 [1.32–4.07]) and 45% lower than Swedish background children (IRR 0.55 [0.31–0.90]). Second generation children with fathers from South-East Asia had the highest incidence of ALL (ASR 4.25 [2.24–8.06]) which was 30% higher than Swedish background children (IRR 1.31 [0.68–2.25]) (Supplementary Table S7). The pattern for ALL by World Bank income level of paternal country of birth mirrored the pattern for leukaemia overall with the lowest incidence in children with fathers from low-income countries (ASR 2.46 [1.77–3.42] and highest for children with fathers from high income countries (4.03 [3.19–5.09]) (Supplementary Table S8). Maternal results were similar (Supplementary Tables S7 and S8).

When lymphoma was broken down by subtype, 361 of the 795 lymphoma cases were Hodgkin lymphoma. ASR for Hodgkin lymphoma was higher in the second generation (1.86 [1.42–2.43], IRR 1.64 [1.21–2.19]) compared to Swedish background (Supplementary Table S6). Small case numbers resulted in ASR with wide confidence intervals when examined by WHO region. Children with mothers from WHO Eastern Mediterranean Region, however, had ASR for Hodgkin lymphoma 1.32 cases per 100,000 person years higher than Swedish background children with a corresponding IRR of 2.01 [1.29–2.97]) (Supplementary Table S7). For World Bank income level, children with parents from middle income countries experienced a slightly higher incidence (0.70–0.83 cases per 100,000 person-years) than Swedish background children (Supplementary Table S8).

No clear pattern emerged for CNS tumour subtype by migrant background (Supplementary Table S6). By WHO parental region of origin, children of parents from WHO Africa Region and low-income countries tended to have lower ASR for CNS tumour subtypes compared to Swedish background children but absolute differences were small (Supplementary Tables S7 and S8).

## Discussion

In this register-based cohort study, we describe 6797 childhood cancer cases occurring in children aged 1–19 born in Sweden between 1990 and 2019. Overall, age-standardised incidence rates were similar across migrant generations. In children with parents from low-income countries, however, we found a 15% lower rate of leukaemia and nearly 50% lower rate of CNS tumours compared to Swedish background children. Given the rarity of childhood cancer, absolute differences in incidence rates were small.

These results mirror global trends in childhood cancer incidence^1^, ^2^, ^3^, ^4^ with GLOBCAN reporting leukaemia incidence in high income countries in children 0–19 in 2022 of 4.7 per 100,000 and 1.6 per 100,000 in low-income countries.^23^ In line with our results, previous studies have also reported higher rate of Hodgkin lymphoma in countries that make up the WHO Eastern Mediterranean Region, with lowest rates in the Nordic countries.^24^ Due to the mandatory, nationwide reporting of childhood cancer in Sweden, our results suggest that there are true differences in childhood cancer incidence between global regions related to disease aetiology. Differences between global regions are likely not solely explained by limited diagnostic capacity and incomplete cancer registration in low-income settings. Although we cannot pinpoint exact aetiology from this nationwide cohort study, given that all children were born in Sweden, the difference in incidence rates in different global regions likely relates to factors that migrate with the parents such as genetic or epigenetic changes, rather than location specific aetiologies such as environmental exposures.

Underlying genetic predisposition to childhood cancer is an active area of research given recent improvements in genetic sequencing technology. In one recent study outlining genetic alterations across a wide range of childhood cancer subtypes (including leukaemias and CNS tumours), 7.6% of cancers were associated with a germline genetic mutation.^25^ This is consistent with a study from the United States which found 8.5% of childhood cancer patients possessed a likely pathogenic mutation.^26^ Studies, however, have yet to compare genetic mutation distribution across diverse backgrounds or compare distribution in different areas of the world.

The underlying aetiology of childhood cancer remains unknown. The only established risk factor for childhood cancer is exposure to ionising radiation.^27^^,^^28^ Research exploring other risk factors is conflicting, with discordant results seen in case control and cohort studies.^27^^,^^29^ Folic acid, for example, was inversely associated and maternal smoking positively associated with childhood brain tumours in several cohort studies but not in case control studies.^29^ Folic acid as an exposure is not likely to explain our observed differences by parental country of origin, since folic acid is less common in mothers from low-income countries.^30^^,^^31^ Maternal smoking is also unlikely to explain the results as smoking is more common in mothers from other Nordic countries, Eastern Europe and Former Yugoslavia countries than Swedish background mothers with Swedish partners.^32^ If smoking were a significant aetiology of childhood cancer, we would expect to see a higher ASR in children with mothers from middle and high income countries, which we did not.

Studies on childhood cancer aetiology and genetics are predominantly conducted in Europe, North America and Asia.^29^ In one recent review and meta-analysis of potential aetiologies for childhood brain tumours, no studies were reported from Africa, potentially limiting the interpretation for global populations^29^ and highlighting the need for studies that incorporate a diverse population and understudied areas.

Unlike prior studies on type 1 diabetes and asthma,^8^^,^^9^ parental length of residence in Sweden does not affect the incidence of childhood cancer in their offspring. One would expect parents who resided in Sweden for a longer period of time to have more similar environmental and societal exposures to Swedish-born parents than newly arrived migrants. The lack of a dose-like response in childhood cancer incidence by parental length of stay in Sweden therefore argues against a cumulative societal or environmental aetiology for childhood cancer.

A major strength of this study is the population-based approach using high-quality national register data, which comprehensively captures the entire Swedish population. Childhood cancer is a rare disease and we are able to capture virtually all cases that occurred in Sweden from 1991 to 2021 in the 1990–2019 birth cohort. This represents a contemporary time period that is directly relevant to children living in Sweden today. In addition, we capture a period of high international migration to Sweden.^33^ Given that Sweden has universal healthcare and national mandatory cancer registration, it is unlikely that underreporting or underdiagnoses explain differences in cancer incidence by migrant background. Moreover, the unique personal identification number allows us to link multiple generations of family members to include both children and grandchildren of migrants in our study. This is the first study on cancer incidence in migrant populations to include the third generation and to take into consideration the heterogeneity of migrant populations by considering migration generation, parental lineages separately, parental length of residence, and World bank income level of parental country of birth.

There are some limitations to our study. We were unable to include children under the age of one year due to incomplete capture of children who die before their first birthday. This subgroup likely experiences different risk factors for childhood cancer compared to older children, such as perinatal exposures. Likewise, our study population included children born and registered in Sweden in order to correctly measure incidence of cancer. However, early migrant children and unregistered children may also have a distinct pattern of cancer incidence which should be addressed in future research. Migrant parents were predominantly recent arrivals to Sweden with median length of stay of 6 years for women and 9 years for men. This relatively short duration of stay may have influenced our analysis of parental length of stay, as parents who had resided in Sweden for 15 or more years likely belong to a different migration cohort. The third generation also represents different migration cohorts from the second generation due to timing of immigration to Sweden. Historically, migrants from Finland made up the majority of immigrants to Sweden.^34^ Between 1970 and 1990, migrants to Sweden largely arrived from Yugoslavia, Turkey and Chile.^35^ In the early 1990s, migration shifted to reflect the fall of the Soviet Union and the Yugoslavian civil war.^36^^,^^37^ Whereas, in 2019, 20% of the foreign-born population was from Syria or Iraq.^38^ To account for this potential heterogeneity, we analysed parental length of stay in five-year increments rather than applying a dichotomous threshold of 10 or 15 years to define “long” duration of stay as has been done previously. Even with this more refined categorisation, however, we did not observe any indication of a step-wise relationship between parental length of stay and childhood cancer incidence. We are unable to rule out that there may be different mechanisms driving low incidence rates in low-income countries and low incidence rates among children born in Sweden with parents originating from low-income countries. For example, healthy migrants to Sweden may have healthier children. Moreover, we may underestimate the incidence of childhood cancers that occur in teenage years as half of the second-generation population in our study was born between 2010 and 2019. This resulted in an average follow up time of 10 years for the second generation. Age standardisation, however, should mitigate any differences in age structure of the populations. Future studies will be needed to follow this group overtime. Moreover, future studies examining subgroups of migrants from low World Bank income level would add further nuance to the body evidence. However, even larger sample sizes are required for such a study.

In conclusion, there were no major disparities in childhood cancer incidence across migrant background. There, however, were notable differences by World Bank income level of parental country of origin. Children with parents from low-income countries had a 15% lower incidence of leukaemia and a 50% lower incidence of CNS tumours compared to Swedish background children, while children with migrant parents from high-income countries experienced a 20–40% higher rate of leukaemia compared to Swedish background children. Our findings in the Swedish context challenge the hypothesis that low incidence of CNS tumours and leukaemia, specifically ALL, in low-income countries is solely due to limited diagnostic capabilities and incomplete cancer registration but rather suggests that genetic or intergenerational environmental factors may also underly these observations.

## Contributors

HLB acquired funding and data as well as supervised the study. HLB and GA had access to the raw data and verified the data. GA carried out the data analysis, interpretation and drafted the original manuscript. GA, EE, SA, HM, HLB contributed to study conception, design, data interpretation, editing the manuscript, and approved the final manuscript. HLB had final responsibility for the decision to submit the manuscript for publication.

## Data sharing statement

The data that support the findings of this study are available from Statistics Sweden and the National Board of Health and Welfare but restrictions apply to the availability of these data. The data were used under ethics approval for the current study, so are not publicly available. Researchers can, after ethical approval, apply for the data from Statistics Sweden and the Swedish National Board of Health and Welfare.

## Use of generative AI technology

An AI assistant was used for code suggestions and debugging. Suggestions were verified before use.

## Declaration of interests

The authors declare no competing interests.
