# Supplementary material for: Childhood cancer incidence by migrant background in Sweden (1991–2021): a nationwide cohort study
Source: Lancet Reg Health Eur. 2026 Feb 20;64:101621. doi: 10.1016/j.lanepe.2026.101621 (PMC12936785; doi:10.1016/j.lanepe.2026.101621)
Supplement: Supplementary Material [file mmc1.pdf]

Supplementary material for:

Childhood cancer incidence by migrant background in Sweden (1991-2021): a nationwide cohort study

Genevieve Allen[1], Elena Extrand[1], Siddartha Aradhya[2], Hanna Mogensen[3], Hannah L Brooke*[1]

1 Medical Epidemiology, Department of Surgical Sciences, Uppsala University, Uppsala, Sweden

2 Stockholm University Demography Unit (SUDA), Department of Sociology, Stockholm University, Stockholm, Sweden

3 Department of Immunology, Genetics and Pathology, Cancer Precision Medicine, Uppsala University, Uppsala, Sweden

***corresponding author: Hannah.brooke@uu.se**

Table of Contents:

Supplementary Methods 2

Supplementary Figure 1 3

Supplementary Figure 2 4

Supplementary Table 1 5

Supplementary Table 2 6

Supplementary Table 3 8

Supplementary Table 4 10

Supplementary Table 5 14

Supplementary Table 6 18

Supplementary Table 7 20

Supplementary Table 8 24

Supplementary Methods

World Bank Country of Birth Income Assessment:

Income values were extracted from the World Bank Historical Classification of incomes available from (https://datatopics.worldbank.org/world-development-indicators/the-world-by-income-and-region.html) based on:

- Year of migration
- For migration year before 1987, from first year of recorded income level (typically 1987 value)
- If there was no immigration year, but the country of birth was consistently in one income class, they were assigned the consistent income class. For example, the USA, Canada, Norway were assigned High-income and Eritrea — Low-income
- The World Bank does not recognize Palestine but does list Gaza and West Bank. Palestine was assigned based on classification of Gaza and West Bank.
- For USSR, Yugoslavia, and Czechoslovakia listed as country of birth but immigration year was after 1991, I assigned upper-middle Income.
- If Kosovo, Serbia, and Montenegro were listed as country of birth but migration occurred before independence and thus the World Bank did not report, they were considered lower-middle income.
- This resulted in only 524 with unknown maternal country of birth income level (mothers who moved from countries with changing income levels and no migration date).

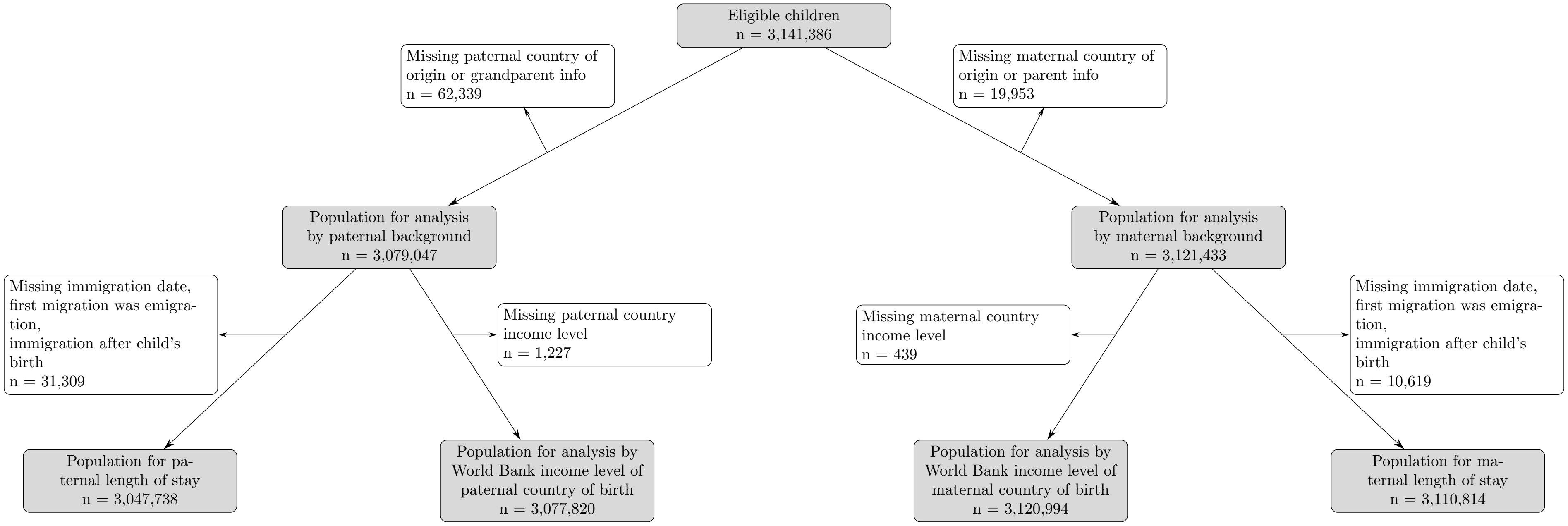

Supplementary Figure 1. Selection of the population for analyses by maternal and paternal background.

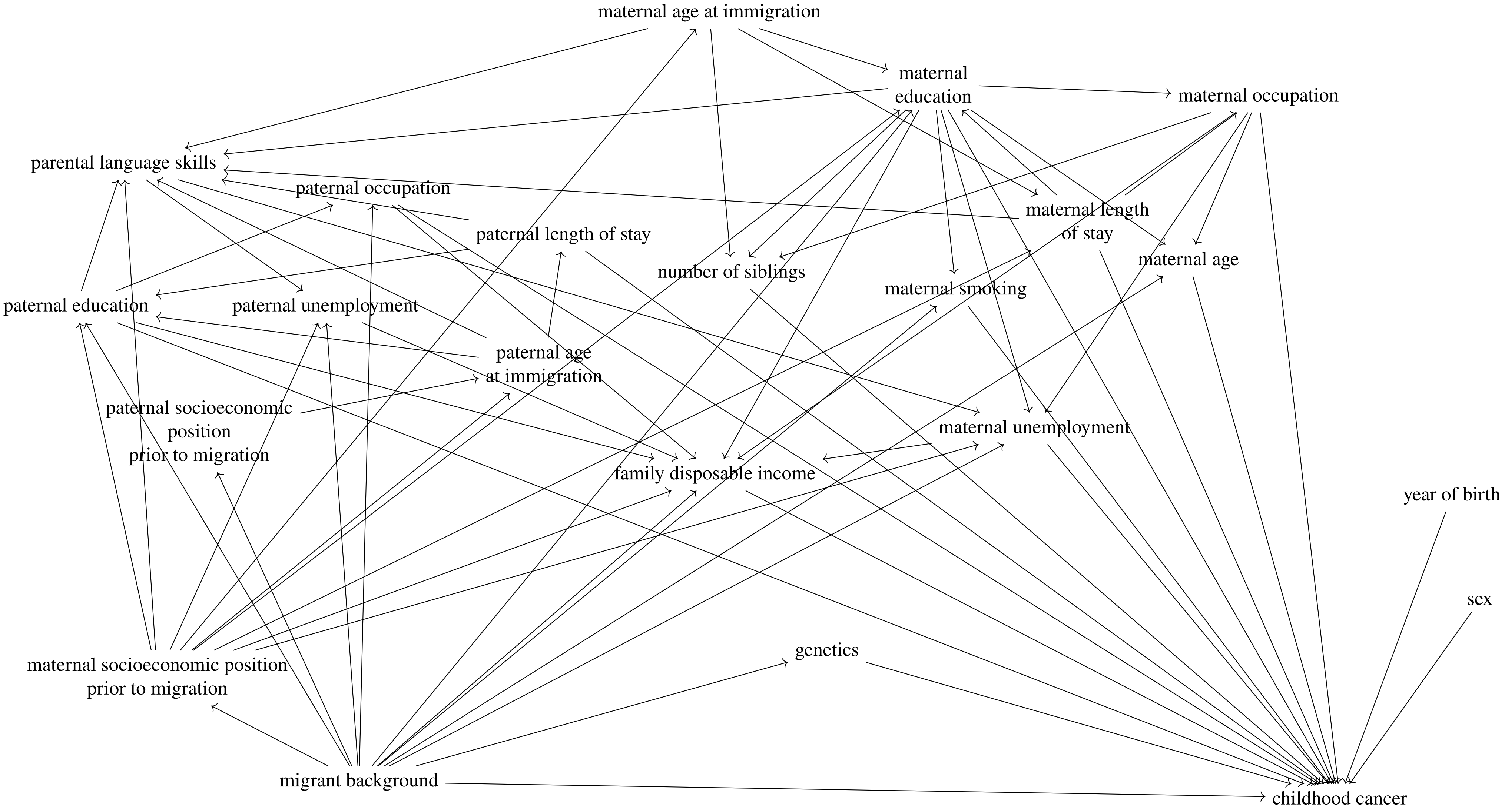

Supplementary Figure 2. Directed acyclic graph to identify covariables for the total effects of migrant background on childhood cancer incidence diagnosed in children born in Sweden 1990-2019.

Supplementary Table 1. Median follow-up time in years for children born in Sweden 1990-2019 by migrant background

Migrant background	Median follow up time (years)	Interquartile range (years)
Swedish background	16·16	8·64 - 19·00
3rd generation	15·50	8·16 - 19·00
2·5 generation	12·92	6·42 - 19·00
2nd generation	9·88	4·66 - 18·47
Overall	14·68	7·42 - 19·00

Supplementary Table 2. Cancer incidence between ages 1 and 19 in the years 1991 through 2021 in Sweden by migrant background

Migrant Background	Sex	Cases	Person-years	ASR per 100,000 persons	95% CI	IRR	95% CI
**I-XII. All Childhood Cancer**							
Swedish background	Both Sexes	4062	23615680	17·18	16·63–17·74	1·00	reference group
Third generation	Both Sexes	1112	6678479	16·86	15·85–17·94	0·97	0·91–1·03
2·5 generation	Both Sexes	793	4714534	16·96	15·74–18·27	0·98	0·91–1·05
Second generation	Both Sexes	830	4852453	17·70	16·40–19·09	0·99	0·92–1·07
Swedish background	Male	2219	12142125	18·20	17·42–19·01	1·00	reference group
Third generation	Male	588	3435609	17·51	16·09–19·06	0·94	0·85–1·03
2·5 generation	Male	392	2418899	16·27	14·63–18·09	0·89	0·80–0·99
Second generation	Male	451	2491473	18·29	16·50–20·28	0·99	0·90–1·10
Swedish background	Female	1843	11473555	16·09	15·34–16·88	1·00	reference group
Third generation	Female	524	3242870	16·17	14·77–17·70	1·01	0·91–1·11
2·5 generation	Female	401	2295635	17·69	15·92–19·65	1·09	0·97–1·21
Second generation	Female	379	2360980	17·07	15·26–19·09	1·00	0·89–1·11
**I. Leukaemias**							
Swedish background	Both Sexes	1179	23615680	4·33	4·08–4·59	1·00	reference group
Third generation	Both Sexes	315	6678479	4·03	3·59–4·52	0·94	0·83–1·06
2·5 generation	Both Sexes	264	4714534	4·84	4·26–5·50	1·09	0·96–1·25
Second generation	Both Sexes	269	4852453	4·72	4·14–5·39	1·06	0·92–1·20
Swedish background	Male	649	12142125	4·75	4·38–5·14	1·00	reference group
Third generation	Male	179	3435609	4·56	3·92–5·32	0·97	0·82–1·14
2·5 generation	Male	141	2418899	5·16	4·33–6·15	1·07	0·89–1·28
Second generation	Male	164	2491473	5·69	4·80–6·74	1·19	1·00–1·41
Swedish background	Female	530	11473555	3·88	3·56–4·24	1·00	reference group
Third generation	Female	136	3242870	3·46	2·90–4·13	0·90	0·74–1·08
2·5 generation	Female	123	2295635	4·51	3·75–5·43	1·12	0·92–1·36
Second generation	Female	105	2360980	3·70	3·00–4·56	0·90	0·73–1·10
**II. Lymphomas**							
Swedish background	Both Sexes	446	23615680	2·22	2·02–2·44	1·00	reference group
Third generation	Both Sexes	138	6678479	2·52	2·12–3·00	1·10	0·91–1·33
2·5 generation	Both Sexes	84	4714534	2·26	1·81–2·84	0·97	0·76–1·22
Second generation	Both Sexes	109	4852453	3·09	2·53–3·78	1·26	1·02–1·55
Swedish background	Male	287	12142125	2·73	2·42–3·07	1·00	reference group
Third generation	Male	92	3435609	3·21	2·60–3·97	1·14	0·90–1·44

abbreviations: ICCC3 - International Classification of Childhood Cancer, third edition, ASR - age-standardized incidence rate, IRR - Incidence rate ratio, CI - Confidence interval; IRR for both sexes calculated from multivariable poisson regression adjusted for sex and year of birth. For sex-specific analyses, models were adjusted for year of birth

Supplementary Table 2. Cancer incidence between ages 1 and 19 in the years 1991 through 2021 in Sweden by migrant background

Migrant Background	Sex	Cases	Person-years	ASR per 100,000 persons	95% CI	IRR	95% CI
2·5 generation	Male	52	2418899	2·54	1·90–3·39	0·93	0·69–1·24
Second generation	Male	59	2491473	2·98	2·26–3·92	1·06	0·79–1·39
Swedish background	Female	159	11473555	1·69	1·44–1·98	1·00	reference group
Third generation	Female	46	3242870	1·79	1·33–2·41	1·03	0·73–1·42
2·5 generation	Female	32	2295635	1·98	1·38–2·83	1·04	0·70–1·50
Second generation	Female	50	2360980	3·22	2·41–4·29	1·63	1·17–2·23
**III. CNS**							
Swedish background	Both Sexes	1074	23615680	4·51	4·24–4·80	1·00	reference group
Third generation	Both Sexes	300	6678479	4·54	4·03–5·11	0·99	0·87–1·12
2·5 generation	Both Sexes	192	4714534	4·21	3·62–4·90	0·90	0·77–1·04
Second generation	Both Sexes	197	4852453	4·22	3·61–4·92	0·90	0·77–1·04
Swedish background	Male	577	12142125	4·50	4·13–4·90	1·00	reference group
Third generation	Male	152	3435609	4·35	3·68–5·13	0·93	0·77–1·11
2·5 generation	Male	81	2418899	3·23	2·57–4·07	0·70	0·55–0·88
Second generation	Male	97	2491473	3·87	3·11–4·82	0·81	0·65–1·00
Swedish background	Female	497	11473555	4·53	4·13–4·96	1·00	reference group
Third generation	Female	148	3242870	4·73	4·00–5·61	1·06	0·88–1·27
2·5 generation	Female	111	2295635	5·25	4·30–6·40	1·13	0·91–1·38
Second generation	Female	100	2360980	4·58	3·69–5·70	1·00	0·80–1·23
**IV-XI. Other Solid Tumours**							
Swedish background	Both Sexes	1342	23615680	6·03	5·70–6·37	1·00	reference group
Third generation	Both Sexes	356	6678479	5·74	5·14–6·40	0·94	0·84–1·06
2·5 generation	Both Sexes	252	4714534	5·60	4·90–6·39	0·95	0·83–1·08
Second generation	Both Sexes	252	4852453	5·62	4·90–6·46	0·93	0·81–1·07
Swedish background	Male	695	12142125	6·14	5·68–6·64	1·00	reference group
Third generation	Male	164	3435609	5·36	4·57–6·30	0·84	0·70–0·99
2·5 generation	Male	117	2418899	5·27	4·34–6·40	0·86	0·70–1·04
Second generation	Male	130	2491473	5·72	4·72–6·94	0·94	0·78–1·13
Swedish background	Female	647	11473555	5·90	5·45–6·40	1·00	reference group
Third generation	Female	192	3242870	6·14	5·29–7·12	1·05	0·89–1·23
2·5 generation	Female	135	2295635	5·95	4·96–7·13	1·05	0·87–1·26
Second generation	Female	122	2360980	5·52	4·52–6·73	0·92	0·76–1·12

abbreviations: ICCC3 - International Classification of Childhood Cancer, third edition, ASR - age-standardized incidence rate, IRR - Incidence rate ratio, CI - Confidence interval; IRR for both sexes calculated from multivariable poisson regression adjusted for sex and year of birth. For sex-specific analyses, models were adjusted for year of birth

Supplementary Table 3. Cancer incidence between ages 1 and 19 in the years 1991 through 2021 in Sweden by maternal and paternal migrant background

		Father						Mother					
**Migrant Background**	**Sex**	**Cases**	**Person-years**	**ASR per 100,000 persons**	**95% CI**	**IRR**	**95% CI**	**Cases**	**Person-years**	**ASR per 100,000 persons**	**95% CI**	**IRR**	**95% CI**
**I-XII. All Childhood Cancer**													
Swedish background	Both Sexes	4882	28519434	17·06	16·57–17·57	1·00	reference group	4934	28899855	17·10	16·61–17·61	1·00	reference group
Third generation	Both Sexes	692	4121257	17·17	15·86–18·58	0·98	0·90–1·06	719	4344624	16·75	15·51–18·10	0·97	0·90–1·05
Second generation	Both Sexes	1233	7367841	17·31	16·28–18·40	0·98	0·92–1·04	1235	7150810	17·52	16·47–18·63	1·01	0·95–1·08
Swedish background	Male	2657	14669360	18·04	17·33–18·77	1·00	reference group	2662	14850761	17·93	17·23–18·66	1·00	reference group
Third generation	Male	363	2115711	17·56	15·74–19·58	0·95	0·85–1·06	367	2234779	16·94	15·20–18·87	0·92	0·82–1·02
Second generation	Male	640	3777449	17·33	15·91–18·87	0·94	0·86–1·02	660	3674193	17·77	16·34–19·34	1·00	0·92–1·09
Swedish background	Female	2225	13850074	16·03	15·35–16·74	1·00	reference group	2272	14049094	16·23	15·54–16·95	1·00	reference group
Third generation	Female	329	2005546	16·76	14·94–18·80	1·02	0·91–1·14	352	2109845	16·56	14·82–18·50	1·03	0·92–1·15
Second generation	Female	593	3590392	17·29	15·83–18·89	1·03	0·94–1·12	575	3476617	17·25	15·76–18·88	1·02	0·93–1·12
**I. Leukaemias**													
Swedish background	Both Sexes	1424	28519434	4·32	4·09–4·56	1·00	reference group	1440	28899855	4·31	4·09–4·55	1·00	reference group
Third generation	Both Sexes	217	4121257	4·47	3·89–5·14	1·04	0·90–1·20	198	4344624	3·98	3·43–4·60	0·90	0·78–1·05
Second generation	Both Sexes	388	7367841	4·58	4·11–5·10	1·01	0·90–1·13	419	7150810	4·94	4·45–5·49	1·13	1·01–1·26
Swedish background	Male	792	14669360	4·77	4·44–5·13	1·00	reference group	792	14850761	4·73	4·40–5·09	1·00	reference group
Third generation	Male	119	2115711	4·91	4·07–5·93	1·03	0·85–1·24	105	2234779	4·18	3·42–5·11	0·87	0·71–1·06
Second generation	Male	223	3777449	5·22	4·52–6·03	1·06	0·91–1·23	247	3674193	5·75	5·01–6·59	1·22	1·06–1·41
Swedish background	Female	632	13850074	3·83	3·53–4·16	1·00	reference group	648	14049094	3·87	3·57–4·19	1·00	reference group
Third generation	Female	98	2005546	4·01	3·25–4·93	1·05	0·85–1·29	93	2109845	3·76	3·03–4·65	0·94	0·75–1·16
Second generation	Female	165	3590392	3·90	3·32–4·60	0·95	0·80–1·13	172	3476617	4·10	3·49–4·82	1·01	0·85–1·20
**II. Lymphomas**													
Swedish background	Both Sexes	534	28519434	2·21	2·03–2·42	1·00	reference group	559	28899855	2·28	2·10–2·49	1·00	reference group
Third generation	Both Sexes	82	4121257	2·47	1·97–3·09	1·08	0·85–1·35	87	4344624	2·50	2·02–3·11	1·05	0·83–1·31
Second generation	Both Sexes	163	7367841	2·95	2·50–3·47	1·24	1·04–1·47	141	7150810	2·66	2·23–3·17	1·07	0·89–1·29
Swedish background	Male	348	14669360	2·74	2·46–3·05	1·00	reference group	359	14850761	2·79	2·51–3·11	1·00	reference group

abbreviations: ICCC3 - International Classification of Childhood Cancer, third edition, ASR - age-standardized incidence rate, IRR - Incidence rate ratio, CI - Confidence interval; IRR for both sexes calculated from multivariable poisson regression adjusted for sex and year of birth. For sex-specific analyses, models were adjusted for year of birth

Supplementary Table 3. Cancer incidence between ages 1 and 19 in the years 1991 through 2021 in Sweden by maternal and paternal migrant background

		Father						Mother					
**Migrant Background**	**Sex**	**Cases**	**Person-years**	**ASR per 100,000 persons**	**95% CI**	**IRR**	**95% CI**	**Cases**	**Person-years**	**ASR per 100,000 persons**	**95% CI**	**IRR**	**95% CI**
Third generation	Male	51	2115711	2·96	2·22–3·93	1·03	0·76–1·37	62	2234779	3·38	2·61–4·38	1·16	0·88–1·51
Second generation	Male	95	3777449	3·11	2·51–3·86	1·11	0·88–1·39	75	3674193	2·50	1·95–3·19	0·89	0·68–1·13
Swedish background	Female	186	13850074	1·66	1·43–1·92	1·00	reference group	200	14049094	1·74	1·51–2·01	1·00	reference group
Third generation	Female	31	2005546	1·96	1·36–2·81	1·17	0·79–1·69	25	2109845	1·58	1·06–2·36	0·85	0·54–1·25
Second generation	Female	68	3590392	2·77	2·16–3·55	1·48	1·11–1·95	66	3476617	2·83	2·21–3·64	1·41	1·06–1·85
**III. CNS**													
Swedish background	Both Sexes	1294	28519434	4·50	4·25–4·76	1·00	reference group	1298	28899855	4·49	4·24–4·75	1·00	reference group
Third generation	Both Sexes	175	4121257	4·43	3·79–5·18	0·94	0·80–1·09	201	4344624	4·66	4·03–5·39	1·03	0·89–1·19
Second generation	Both Sexes	300	7367841	4·25	3·75–4·80	0·90	0·79–1·02	290	7150810	4·17	3·68–4·74	0·91	0·80–1·03
Swedish background	Male	683	14669360	4·43	4·09–4·79	1·00	reference group	686	14850761	4·41	4·08–4·76	1·00	reference group
Third generation	Male	92	2115711	4·24	3·42–5·26	0·93	0·74–1·15	93	2234779	4·11	3·32–5·09	0·90	0·72–1·11
Second generation	Male	135	3777449	3·59	2·99–4·30	0·76	0·63–0·91	142	3674193	3·70	3·09–4·43	0·83	0·69–0·99
Swedish background	Female	611	13850074	4·58	4·21–4·97	1·00	reference group	612	14049094	4·57	4·21–4·96	1·00	reference group
Third generation	Female	83	2005546	4·63	3·70–5·81	0·94	0·75–1·18	108	2109845	5·24	4·29–6·39	1·18	0·96–1·44
Second generation	Female	165	3590392	4·94	4·18–5·83	1·06	0·89–1·26	148	3476617	4·67	3·91–5·58	1·00	0·83–1·19
**IV-XI. Other Solid Tumours**													
Swedish background	Both Sexes	1607	28519434	5·96	5·66–6·27	1·00	reference group	1614	28899855	5·95	5·65–6·26	1·00	reference group
Third generation	Both Sexes	216	4121257	5·73	4·98–6·60	0·93	0·81–1·08	231	4344624	5·58	4·87–6·40	0·96	0·83–1·10
Second generation	Both Sexes	379	7367841	5·51	4·93–6·16	0·93	0·83–1·04	381	7150810	5·68	5·09–6·35	0·97	0·87–1·09
Swedish background	Male	823	14669360	6·03	5·61–6·48	1·00	reference group	813	14850761	5·92	5·51–6·36	1·00	reference group
Third generation	Male	99	2115711	5·33	4·33–6·56	0·84	0·68–1·03	107	2234779	5·26	4·31–6·43	0·88	0·72–1·07
Second generation	Male	186	3777449	5·38	4·59–6·31	0·90	0·76–1·05	194	3674193	5·75	4·92–6·72	0·99	0·84–1·16
Swedish background	Female	784	13850074	5·88	5·46–6·33	1·00	reference group	801	14049094	5·97	5·55–6·42	1·00	reference group
Third generation	Female	117	2005546	6·16	5·09–7·46	1·03	0·85–1·25	124	2109845	5·92	4·91–7·14	1·03	0·85–1·24
Second generation	Female	193	3590392	5·65	4·83–6·59	0·96	0·81–1·12	187	3476617	5·61	4·79–6·57	0·95	0·81–1·11

abbreviations: ICCC3 - International Classification of Childhood Cancer, third edition, ASR - age-standardized incidence rate, IRR - Incidence rate ratio, CI - Confidence interval; IRR for both sexes calculated from multivariable poisson regression adjusted for sex and year of birth. For sex-specific analyses, models were adjusted for year of birth

Supplementary Table 4. Cancer incidence between ages 1 and 19 in the years 1991 through 2021 in Sweden by maternal and paternal length of stay

		Father						Mother					
Migrant Background	Sex	Cases	Person-years	ASR per 100,000 persons	95% CI	IRR	95% CI	Cases	Person-years	ASR per 100,000 persons	95% CI	IRR	95% CI
**I-XII. All Childhood Cancer**													
Swedish background	Both Sexes	4882	28519434·0	17·06	16·57–17·57	1·00	reference group	4934	28899855·0	17·10	16·61–17·61	1·00	reference group
Third generation	Both Sexes	692	4121257·3	17·17	15·86–18·58	0·98	0·91–1·06	719	4344623·5	16·75	15·51–18·10	0·97	0·90–1·05
Second generation - 15+ years	Both Sexes	386	2130010·7	19·04	17·05–21·26	1·06	0·95–1·17	322	1684619·8	18·79	16·64–21·21	1·12	1·00–1·25
Second generation - 10-14 years	Both Sexes	172	1084313·9	15·50	13·19–18·21	0·93	0·79–1·07	141	844285·7	15·75	13·13–18·89	0·98	0·82–1·15
Second generation - 5-9 years	Both Sexes	281	1605427·3	18·03	15·87–20·47	1·02	0·90–1·15	262	1507398·6	17·94	15·69–20·51	1·02	0·90–1·15
Second generation - less than 5 years	Both Sexes	348	2148479·4	17·15	15·25–19·28	0·95	0·85–1·05	485	2936259·7	17·41	15·77–19·23	0·97	0·88–1·06
Swedish background	Male	2657	14669360·3	18·04	17·33–18·77	1·00	reference group	2662	14850760·5	17·93	17·23–18·66	1·00	reference group
Third generation	Male	363	2115711·4	17·56	15·74–19·58	0·95	0·85–1·06	367	2234778·7	16·94	15·20–18·87	0·92	0·82–1·02
Second generation - 15+ years	Male	199	1088698·5	19·23	16·49–22·44	1·01	0·87–1·16	173	862384·1	19·47	16·50–22·98	1·12	0·96–1·30
Second generation - 10-14 years	Male	103	558154·0	17·52	14·24–21·57	1·02	0·83–1·24	74	436102·6	15·24	11·87–19·57	0·95	0·75–1·19
Second generation - 5-9 years	Male	140	823713·5	18·12	15·13–21·69	0·94	0·79–1·11	135	774076·1	18·05	14·97–21·77	0·97	0·82–1·15
Second generation - less than 5 years	Male	169	1103466·3	15·30	12·93–18·11	0·85	0·72–0·99	259	1510665·4	17·30	15·11–19·81	0·96	0·84–1·09
Swedish background	Female	2225	13850073·7	16·03	15·35–16·74	1·00	reference group	2272	14049094·5	16·23	15·54–16·95	1·00	reference group
Third generation	Female	329	2005545·9	16·76	14·94–18·80	1·02	0·91–1·14	352	2109844·8	16·56	14·82–18·50	1·03	0·92–1·15
Second generation - 15+ years	Female	187	1041312·2	18·84	16·08–22·07	1·11	0·96–1·29	149	822235·7	18·07	15·12–21·59	1·12	0·94–1·32
Second generation - 10-14 years	Female	69	526160·0	13·35	10·35–17·23	0·81	0·63–1·03	67	408183·1	16·28	12·50–21·21	1·01	0·79–1·28
Second generation - 5-9 years	Female	141	781713·8	17·93	14·98–21·47	1·12	0·94–1·32	127	733322·5	17·82	14·71–21·59	1·07	0·89–1·27
Second generation - less than 5 years	Female	179	1045013·1	19·10	16·24–22·47	1·06	0·91–1·24	226	1425594·3	17·53	15·16–20·27	0·98	0·85–1·12
**I. Leukaemias**													
Swedish background	Both Sexes	1424	28519434·0	4·32	4·09–4·56	1·00	reference group	1440	28899855·0	4·31	4·09–4·55	1·00	reference group
Third generation	Both Sexes	217	4121257·3	4·47	3·89–5·14	1·04	0·90–1·20	198	4344623·5	3·98	3·43–4·60	0·90	0·78–1·05
Second generation - 15+ years	Both Sexes	125	2130010·7	5·48	4·51–6·65	1·12	0·93–1·34	114	1684619·8	5·47	4·49–6·67	1·30	1·07–1·56
Second generation - 10-14 years	Both Sexes	48	1084313·9	3·82	2·82–5·17	0·85	0·63–1·13	54	844285·7	5·25	3·94–6·99	1·23	0·92–1·59

abbreviations: ICCC3 - International Classification of Childhood Cancer, third edition, ASR - age-standardized incidence rate, IRR - Incidence rate ratio, CI - Confidence interval; IRR for both sexes calculated from multivariable poisson regression adjusted for sex and year of birth. For sex-specific analyses, models were adjusted for year of birth alone.

Supplementary Table 4. Cancer incidence between ages 1 and 19 in the years 1991 through 2021 in Sweden by maternal and paternal length of stay

		Father						Mother					
**Migrant Background**	**Sex**	**Cases**	**Person-years**	**ASR per 100,000 persons**	**95% CI**	**IRR**	**95% CI**	**Cases**	**Person-years**	**ASR per 100,000 persons**	**95% CI**	**IRR**	**95% CI**
Second generation - 5-9 years	Both Sexes	92	1605427·3	5·13	4·11–6·39	1·10	0·88–1·35	89	1507398·6	5·03	4·00–6·33	1·12	0·90–1·38
Second generation - less than 5 years	Both Sexes	111	2148479·4	4·07	3·33–4·98	0·99	0·81–1·20	156	2936259·7	4·63	3·89–5·51	1·02	0·86–1·20
Swedish background	Male	792	14669360·3	4·77	4·44–5·13	1·00	reference group	792	14850760·5	4·73	4·40–5·09	1·00	reference group
Third generation	Male	119	2115711·4	4·91	4·07–5·93	1·03	0·85–1·25	105	2234778·7	4·18	3·42–5·11	0·87	0·71–1·06
Second generation - 15+ years	Male	67	1088698·5	5·89	4·51–7·68	1·10	0·85–1·40	58	862384·1	5·56	4·20–7·36	1·22	0·92–1·58
Second generation - 10-14 years	Male	32	558154·0	5·10	3·50–7·42	1·03	0·71–1·45	36	436102·6	7·05	4·97–10·02	1·50	1·05–2·06
Second generation - 5-9 years	Male	54	823713·5	6·09	4·55–8·14	1·18	0·88–1·53	46	774076·1	5·07	3·68–6·99	1·07	0·78–1·43
Second generation - less than 5 years	Male	64	1103466·3	4·52	3·46–5·90	1·04	0·80–1·33	102	1510665·4	5·83	4·69–7·24	1·22	0·99–1·50
Swedish background	Female	632	13850073·7	3·83	3·53–4·16	1·00	reference group	648	14049094·5	3·87	3·57–4·19	1·00	reference group
Third generation	Female	98	2005545·9	4·01	3·25–4·93	1·05	0·85–1·29	93	2109844·8	3·76	3·03–4·65	0·94	0·75–1·16
Second generation - 15+ years	Female	58	1041312·2	5·05	3·81–6·70	1·14	0·86–1·48	56	822235·7	5·38	4·06–7·12	1·39	1·05–1·81
Second generation - 10-14 years	Female	16	526160·0	2·45	1·47–4·08	0·63	0·37–1·01	18	408183·1	3·32	2·01–5·47	0·90	0·54–1·39
Second generation - 5-9 years	Female	38	781713·8	4·12	2·94–5·77	1·01	0·71–1·38	43	733322·5	4·99	3·59–6·93	1·19	0·86–1·59
Second generation - less than 5 years	Female	47	1045013·1	3·60	2·65–4·89	0·93	0·68–1·24	54	1425594·3	3·35	2·51–4·48	0·77	0·58–1·01
**II. Lymphomas**													
Swedish background	Both Sexes	534	28519434·0	2·21	2·03–2·42	1·00	reference group	559	28899855·0	2·28	2·10–2·49	1·00	reference group
Third generation	Both Sexes	82	4121257·3	2·47	1·97–3·09	1·08	0·85–1·35	87	4344623·5	2·50	2·02–3·11	1·05	0·83–1·31
Second generation - 15+ years	Both Sexes	53	2130010·7	3·34	2·50–4·45	1·41	1·05–1·86	34	1684619·8	2·69	1·88–3·85	1·10	0·76–1·53
Second generation - 10-14 years	Both Sexes	20	1084313·9	2·21	1·40–3·49	1·03	0·64–1·57	13	844285·7	1·96	1·09–3·52	0·84	0·46–1·40
Second generation - 5-9 years	Both Sexes	37	1605427·3	3·18	2·26–4·45	1·30	0·91–1·78	32	1507398·6	2·96	2·05–4·27	1·17	0·80–1·64
Second generation - less than 5 years	Both Sexes	45	2148479·4	2·85	2·09–3·90	1·17	0·85–1·57	57	2936259·7	2·70	2·05–3·57	1·06	0·80–1·38
Swedish background	Male	348	14669360·3	2·74	2·46–3·05	1·00	reference group	359	14850760·5	2·79	2·51–3·11	1·00	reference group
Third generation	Male	51	2115711·4	2·96	2·22–3·93	1·03	0·76–1·37	62	2234778·7	3·38	2·61–4·38	1·16	0·88–1·51
Second generation - 15+ years	Male	30	1088698·5	3·56	2·42–5·23	1·23	0·83–1·76	19	862384·1	2·59	1·59–4·21	0·96	0·58–1·48
Second generation - 10-14 years	Male	15	558154·0	3·04	1·79–5·16	1·19	0·68–1·92	5	436102·6	1·47	0·57–3·80	0·50	0·18–1·08

abbreviations: ICCC3 - International Classification of Childhood Cancer, third edition, ASR - age-standardized incidence rate, IRR - Incidence rate ratio, CI - Confidence interval; IRR for both sexes calculated from multivariable poisson regression adjusted for sex and year of birth. For sex-specific analyses, models were adjusted for year of birth alone.

Supplementary Table 4. Cancer incidence between ages 1 and 19 in the years 1991 through 2021 in Sweden by maternal and paternal length of stay

		Father						Mother					
Migrant Background	Sex	Cases	Person-years	ASR per 100,000 persons	95% CI	IRR	95% CI	Cases	Person-years	ASR per 100,000 persons	95% CI	IRR	95% CI
Second generation - 5-9 years	Male	19	823713·5	3·03	1·88–4·89	1·02	0·62–1·57	15	774076·1	2·63	1·53–4·52	0·85	0·48–1·37
Second generation - less than 5 years	Male	25	1103466·3	2·60	1·70–4·00	1·00	0·65–1·46	32	1510665·4	2·56	1·75–3·74	0·92	0·63–1·30
Swedish background	Female	186	13850073·7	1·66	1·43–1·92	1·00	reference group	200	14049094·5	1·74	1·51–2·01	1·00	reference group
Third generation	Female	31	2005545·9	1·96	1·36–2·81	1·17	0·79–1·69	25	2109844·8	1·58	1·06–2·36	0·85	0·54–1·25
Second generation - 15+ years	Female	23	1041312·2	3·11	2·01–4·79	1·75	1·11–2·65	15	822235·7	2·80	1·66–4·72	1·36	0·77–2·22
Second generation - 10-14 years	Female	5	526160·0	1·33	0·54–3·25	0·75	0·27–1·63	8	408183·1	2·47	1·17–5·23	1·47	0·66–2·78
Second generation - 5-9 years	Female	18	781713·8	3·33	2·06–5·37	1·81	1·08–2·86	17	733322·5	3·31	2·00–5·46	1·75	1·03–2·79
Second generation - less than 5 years	Female	20	1045013·1	3·11	1·98–4·89	1·49	0·91–2·31	25	1425594·3	2·85	1·91–4·27	1·31	0·84–1·94
**III. CNS**													
Swedish background	Both Sexes	1294	28519434·0	4·50	4·25–4·76	1·00	reference group	1298	28899855·0	4·49	4·24–4·75	1·00	reference group
Third generation	Both Sexes	175	4121257·3	4·43	3·79–5·18	0·94	0·80–1·09	201	4344623·5	4·66	4·03–5·39	1·03	0·89–1·19
Second generation - 15+ years	Both Sexes	99	2130010·7	4·78	3·86–5·93	1·03	0·83–1·26	70	1684619·8	3·92	3·03–5·08	0·93	0·72–1·17
Second generation - 10-14 years	Both Sexes	41	1084313·9	3·63	2·62–5·02	0·84	0·60–1·12	36	844285·7	4·17	2·92–5·95	0·95	0·67–1·31
Second generation - 5-9 years	Both Sexes	63	1605427·3	3·95	3·02–5·15	0·87	0·67–1·11	53	1507398·6	3·55	2·64–4·76	0·79	0·59–1·02
Second generation - less than 5 years	Both Sexes	86	2148479·4	4·57	3·62–5·78	0·88	0·71–1·09	126	2936259·7	4·76	3·92–5·78	0·96	0·79–1·15
Swedish background	Male	683	14669360·3	4·43	4·09–4·79	1·00	reference group	686	14850760·5	4·41	4·08–4·76	1·00	reference group
Third generation	Male	92	2115711·4	4·24	3·42–5·26	0·93	0·74–1·15	93	2234778·7	4·11	3·32–5·09	0·90	0·72–1·11
Second generation - 15+ years	Male	49	1088698·5	4·40	3·24–5·96	0·95	0·70–1·26	39	862384·1	3·94	2·80–5·55	0·97	0·69–1·32
Second generation - 10-14 years	Male	23	558154·0	3·82	2·49–5·85	0·88	0·56–1·29	13	436102·6	2·85	1·56–5·20	0·64	0·35–1·06
Second generation - 5-9 years	Male	29	823713·5	3·66	2·47–5·42	0·75	0·50–1·06	28	774076·1	3·49	2·33–5·23	0·77	0·52–1·10
Second generation - less than 5 years	Male	30	1103466·3	2·97	2·00–4·42	0·58	0·39–0·82	58	1510665·4	3·93	2·96–5·20	0·82	0·62–1·06
Swedish background	Female	611	13850073·7	4·58	4·21–4·97	1·00	reference group	612	14049094·5	4·57	4·21–4·96	1·00	reference group
Third generation	Female	83	2005545·9	4·63	3·70–5·81	0·94	0·75–1·18	108	2109844·8	5·24	4·29–6·39	1·18	0·96–1·44
Second generation - 15+ years	Female	50	1041312·2	5·19	3·83–7·03	1·11	0·82–1·47	31	822235·7	3·91	2·65–5·77	0·88	0·60–1·25
Second generation - 10-14 years	Female	18	526160·0	3·44	2·09–5·65	0·79	0·48–1·22	23	408183·1	5·58	3·59–8·67	1·32	0·85–1·96

abbreviations: ICCC3 - International Classification of Childhood Cancer, third edition, ASR - age-standardized incidence rate, IRR - Incidence rate ratio, CI - Confidence interval; IRR for both sexes calculated from multivariable poisson regression adjusted for sex and year of birth. For sex-specific analyses, models were adjusted for year of birth alone.

Supplementary Table 4. Cancer incidence between ages 1 and 19 in the years 1991 through 2021 in Sweden by maternal and paternal length of stay

		Father						Mother					
Migrant Background	Sex	Cases	Person-years	ASR per 100,000 persons	95% CI	IRR	95% CI	Cases	Person-years	ASR per 100,000 persons	95% CI	IRR	95% CI
Second generation - 5-9 years	Female	34	781713·8	4·25	2·95–6·11	1·01	0·70–1·40	25	733322·5	3·61	2·35–5·54	0·80	0·52–1·17
Second generation - less than 5 years	Female	56	1045013·1	6·26	4·68–8·37	1·24	0·93–1·61	68	1425594·3	5·65	4·33–7·36	1·12	0·86–1·43
**IV-XI. Other Solid Tumours**													
Swedish background	Both Sexes	1607	28519434·0	5·96	5·66–6·27	1·00	reference group	1614	28899855·0	5·95	5·65–6·26	1·00	reference group
Third generation	Both Sexes	216	4121257·3	5·73	4·98–6·60	0·94	0·81–1·08	231	4344623·5	5·58	4·87–6·40	0·96	0·83–1·10
Second generation - 15+ years	Both Sexes	107	2130010·7	5·37	4·35–6·64	0·91	0·74–1·10	103	1684619·8	6·59	5·31–8·17	1·12	0·91–1·36
Second generation - 10-14 years	Both Sexes	63	1084313·9	5·84	4·46–7·66	1·05	0·81–1·33	38	844285·7	4·38	3·07–6·23	0·82	0·59–1·12
Second generation - 5-9 years	Both Sexes	88	1605427·3	5·74	4·57–7·22	0·99	0·79–1·22	87	1507398·6	6·37	5·05–8·02	1·06	0·85–1·30
Second generation - less than 5 years	Both Sexes	106	2148479·4	5·65	4·58–6·98	0·89	0·73–1·08	144	2936259·7	5·27	4·40–6·32	0·90	0·75–1·06
Swedish background	Male	823	14669360·3	6·03	5·61–6·48	1·00	reference group	813	14850760·5	5·92	5·51–6·36	1·00	reference group
Third generation	Male	99	2115711·4	5·33	4·33–6·56	0·84	0·68–1·03	107	2234778·7	5·26	4·31–6·43	0·88	0·72–1·07
Second generation - 15+ years	Male	52	1088698·5	5·31	3·92–7·21	0·88	0·65–1·15	56	862384·1	7·17	5·35–9·59	1·22	0·92–1·59
Second generation - 10-14 years	Male	33	558154·0	5·57	3·83–8·09	1·08	0·75–1·50	20	436102·6	3·87	2·39–6·27	0·86	0·54–1·31
Second generation - 5-9 years	Male	38	823713·5	5·34	3·79–7·53	0·84	0·60–1·15	46	774076·1	6·87	5·00–9·44	1·12	0·82–1·49
Second generation - less than 5 years	Male	50	1103466·3	5·20	3·82–7·08	0·83	0·61–1·09	66	1510665·4	4·92	3·77–6·43	0·82	0·63–1·05
Swedish background	Female	784	13850073·7	5·88	5·46–6·33	1·00	reference group	801	14049094·5	5·97	5·55–6·42	1·00	reference group
Third generation	Female	117	2005545·9	6·16	5·09–7·46	1·03	0·85–1·25	124	2109844·8	5·92	4·91–7·14	1·03	0·85–1·24
Second generation - 15+ years	Female	55	1041312·2	5·44	4·06–7·28	0·94	0·71–1·23	47	822235·7	5·98	4·35–8·23	1·01	0·74–1·34
Second generation - 10-14 years	Female	30	526160·0	6·14	4·16–9·06	1·02	0·69–1·43	18	408183·1	4·92	2·96–8·18	0·78	0·47–1·21
Second generation - 5-9 years	Female	50	781713·8	6·17	4·55–8·36	1·14	0·85–1·50	41	733322·5	5·84	4·17–8·17	0·99	0·71–1·34
Second generation - less than 5 years	Female	56	1045013·1	6·13	4·59–8·19	0·95	0·72–1·24	78	1425594·3	5·64	4·40–7·22	0·97	0·76–1·22

abbreviations: ICCC3 - International Classification of Childhood Cancer, third edition, ASR - age-standardized incidence rate, IRR - Incidence rate ratio, CI - Confidence interval; IRR for both sexes calculated from multivariable poisson regression adjusted for sex and year of birth. For sex-specific analyses, models were adjusted for year of birth alone.

Supplementary Table 5. Cancer incidence between ages 1 and 19 in the years 1991 through 2021 in Sweden by World Bank income level of maternal and paternal country of birth

		Father						Mother					
Migrant Background	Sex	Cases	Person-years	ASR per 100,000 persons	95% CI	IRR	95% CI	Cases	Person-years	ASR per 100,000 persons	95% CI	IRR	95% CI
**I-XII. All Childhood Cancer**													
Swedish background	Both Sexes	4882	28519434·0	17·06	16·57–17·57	1·00	reference group	4934	28899855·0	17·10	16·61–17·61	1·00	reference group
Third generation	Both Sexes	692	4121257·3	17·17	15·86–18·58	0·98	0·90–1·06	719	4344623·5	16·75	15·51–18·10	0·97	0·90–1·05
Second generation - high income	Both Sexes	284	1612161·2	18·28	16·16–20·68	1·03	0·91–1·16	253	1401801·2	17·11	15·01–19·51	1·06	0·93–1·20
Second generation - middle income	Both Sexes	767	4318563·3	18·09	16·73–19·56	1·04	0·96–1·12	789	4330874·5	18·82	17·41–20·33	1·07	0·99–1·15
Second generation - low income	Both Sexes	179	1420561·7	13·71	11·62–16·16	0·73	0·63–0·85	192	1412724·4	14·22	12·10–16·71	0·80	0·69–0·92
Swedish background	Male	2657	14669360·3	18·04	17·33–18·77	1·00	reference group	2662	14850760·5	17·93	17·23–18·66	1·00	reference group
Third generation	Male	363	2115711·4	17·56	15·74–19·58	0·95	0·85–1·06	367	2234778·7	16·94	15·20–18·87	0·92	0·82–1·02
Second generation - high income	Male	143	824792·1	17·68	14·85–21·05	0·96	0·81–1·13	141	720316·7	18·40	15·43–21·93	1·09	0·92–1·29
Second generation - middle income	Male	398	2215400·9	18·06	16·20–20·13	0·99	0·89–1·10	417	2224164·0	18·74	16·84–20·85	1·05	0·94–1·16
Second generation - low income	Male	97	728913·2	14·56	11·64–18·21	0·74	0·60–0·90	101	726882·3	14·25	11·42–17·77	0·78	0·63–0·94
Swedish background	Female	2225	13850073·7	16·03	15·35–16·74	1·00	reference group	2272	14049094·5	16·23	15·54–16·95	1·00	reference group
Third generation	Female	329	2005545·9	16·76	14·94–18·80	1·02	0·91–1·14	352	2109844·8	16·56	14·82–18·50	1·03	0·92–1·15
Second generation - high income	Female	141	787369·1	18·91	15·87–22·52	1·11	0·94–1·32	112	681484·5	15·76	12·94–19·19	1·02	0·84–1·22
Second generation - middle income	Female	369	2103162·4	18·12	16·19–20·28	1·09	0·97–1·21	372	2106710·5	18·90	16·89–21·15	1·09	0·97–1·21
Second generation - low income	Female	82	691648·5	12·80	10·03–16·35	0·73	0·58–0·91	91	685842·1	14·18	11·20–17·96	0·82	0·66–1·00
**I. Leukaemias**													
Swedish background	Both Sexes	1424	28519434·0	4·32	4·09–4·56	1·00	reference group	1440	28899855·0	4·31	4·09–4·55	1·00	reference group
Third generation	Both Sexes	217	4121257·3	4·47	3·89–5·14	1·04	0·90–1·20	198	4344623·5	3·98	3·43–4·60	0·90	0·78–1·04
Second generation - high income	Both Sexes	96	1612161·2	5·46	4·42–6·75	1·19	0·96–1·45	99	1401801·2	5·88	4·78–7·24	1·42	1·16–1·74
Second generation - middle income	Both Sexes	234	4318563·3	4·56	3·96–5·25	1·03	0·90–1·19	259	4330874·5	5·09	4·45–5·83	1·14	0·99–1·30
Second generation - low income	Both Sexes	58	1420561·7	3·66	2·76–4·85	0·77	0·58–0·99	61	1412724·4	3·68	2·79–4·87	0·81	0·62–1·04
Swedish background	Male	792	14669360·3	4·77	4·44–5·13	1·00	reference group	792	14850760·5	4·73	4·40–5·09	1·00	reference group
Third generation	Male	119	2115711·4	4·91	4·07–5·93	1·03	0·85–1·24	105	2234778·7	4·18	3·42–5·11	0·87	0·71–1·06

abbreviations: ICCC3 - International Classification of Childhood Cancer, third edition, ASR - age-standardized incidence rate, IRR - Incidence rate ratio, CI - Confidence interval; IRR for both sexes calculated from multivariable poisson regression adjusted for sex and year of birth. For sex-specific analyses, models were adjusted for year of birth alone.

Supplementary Table 5. Cancer incidence between ages 1 and 19 in the years 1991 through 2021 in Sweden by World Bank income level of maternal and paternal country of birth

		Father						Mother					
Migrant Background	Sex	Cases	Person-years	ASR per 100,000 persons	95% CI	IRR	95% CI	Cases	Person-years	ASR per 100,000 persons	95% CI	IRR	95% CI
Second generation - high income	Male	53	824792·1	6·03	4·52–8·03	1·19	0·89–1·55	58	720316·7	6·76	5·16–8·85	1·52	1·15–1·96
Second generation - middle income	Male	140	2215400·9	5·42	4·52–6·51	1·13	0·94–1·35	153	2224164·0	5·99	5·02–7·15	1·24	1·04–1·47
Second generation - low income	Male	30	728913·2	3·69	2·48–5·50	0·73	0·49–1·03	36	726882·3	4·21	2·93–6·05	0·88	0·62–1·22
Swedish background	Female	632	13850073·7	3·83	3·53–4·16	1·00	reference group	648	14049094·5	3·87	3·57–4·19	1·00	reference group
Third generation	Female	98	2005545·9	4·01	3·25–4·93	1·05	0·85–1·29	93	2109844·8	3·76	3·03–4·65	0·94	0·75–1·16
Second generation - high income	Female	43	787369·1	4·87	3·56–6·66	1·19	0·86–1·60	41	681484·5	4·95	3·58–6·85	1·31	0·94–1·77
Second generation - middle income	Female	94	2103162·4	3·65	2·94–4·54	0·92	0·74–1·14	106	2106710·5	4·15	3·38–5·10	1·02	0·82–1·24
Second generation - low income	Female	28	691648·5	3·62	2·43–5·41	0·82	0·55–1·17	25	685842·1	3·13	2·03–4·83	0·72	0·47–1·05
**II. Lymphomas**													
Swedish background	Both Sexes	534	28519434·0	2·21	2·03–2·42	1·00	reference group	559	28899855·0	2·28	2·10–2·49	1·00	reference group
Third generation	Both Sexes	82	4121257·3	2·47	1·97–3·09	1·08	0·85–1·35	87	4344623·5	2·50	2·02–3·11	1·05	0·83–1·31
Second generation - high income	Both Sexes	36	1612161·2	2·80	2·00–3·94	1·20	0·84–1·65	22	1401801·2	1·89	1·22–2·92	0·80	0·51–1·20
Second generation - middle income	Both Sexes	108	4318563·3	3·31	2·71–4·05	1·41	1·14–1·73	99	4330874·5	3·15	2·56–3·89	1·26	1·01–1·55
Second generation - low income	Both Sexes	18	1420561·7	1·87	1·14–3·06	0·73	0·44–1·13	19	1412724·4	1·88	1·15–3·08	0·75	0·46–1·16
Swedish background	Male	348	14669360·3	2·74	2·46–3·05	1·00	reference group	359	14850760·5	2·79	2·51–3·11	1·00	reference group
Third generation	Male	51	2115711·4	2·96	2·22–3·93	1·03	0·76–1·37	62	2234778·7	3·38	2·61–4·38	1·16	0·88–1·51
Second generation - high income	Male	21	824792·1	3·01	1·92–4·69	1·07	0·67–1·63	16	720316·7	2·57	1·54–4·30	0·91	0·53–1·45
Second generation - middle income	Male	63	2215400·9	3·50	2·68–4·57	1·26	0·96–1·64	50	2224164·0	2·79	2·07–3·78	0·98	0·72–1·31
Second generation - low income	Male	10	728913·2	1·81	0·93–3·52	0·62	0·31–1·10	8	726882·3	1·16	0·54–2·48	0·49	0·22–0·92
Swedish background	Female	186	13850073·7	1·66	1·43–1·92	1·00	reference group	200	14049094·5	1·74	1·51–2·01	1·00	reference group
Third generation	Female	31	2005545·9	1·96	1·36–2·81	1·17	0·79–1·69	25	2109844·8	1·58	1·06–2·36	0·85	0·54–1·26
Second generation - high income	Female	15	787369·1	2·59	1·54–4·38	1·42	0·80–2·32	6	681484·5	1·17	0·52–2·64	0·61	0·24–1·26
Second generation - middle income	Female	45	2103162·4	3·11	2·29–4·22	1·69	1·20–2·32	49	2106710·5	3·53	2·64–4·73	1·75	1·27–2·38
Second generation - low income	Female	8	691648·5	1·93	0·93–3·99	0·93	0·42–1·77	11	685842·1	2·65	1·41–4·96	1·23	0·63–2·16
**III. CNS**													

abbreviations: ICCC3 - International Classification of Childhood Cancer, third edition, ASR - age-standardized incidence rate, IRR - Incidence rate ratio, CI - Confidence interval; IRR for both sexes calculated from multivariable poisson regression adjusted for sex and year of birth. For sex-specific analyses, models were adjusted for year of birth alone.

Supplementary Table 5. Cancer incidence between ages 1 and 19 in the years 1991 through 2021 in Sweden by World Bank income level of maternal and paternal country of birth

		Father						Mother					
Migrant Background	Sex	Cases	Person-years	ASR per 100,000 persons	95% CI	IRR	95% CI	Cases	Person-years	ASR per 100,000 persons	95% CI	IRR	95% CI
Swedish background	Both Sexes	1294	28519434·0	4·50	4·25–4·76	1·00	reference group	1298	28899855·0	4·49	4·24–4·75	1·00	reference group
Third generation	Both Sexes	175	4121257·3	4·43	3·79–5·18	0·94	0·80–1·09	201	4344623·5	4·66	4·03–5·39	1·03	0·89–1·19
Second generation - high income	Both Sexes	72	1612161·2	4·69	3·67–5·99	0·98	0·77–1·24	56	1401801·2	3·89	2·94–5·14	0·89	0·67–1·15
Second generation - middle income	Both Sexes	194	4318563·3	4·62	3·96–5·38	0·99	0·85–1·15	200	4330874·5	4·78	4·10–5·57	1·03	0·89–1·20
Second generation - low income	Both Sexes	34	1420561·7	2·57	1·76–3·76	0·53	0·37–0·73	34	1412724·4	2·64	1·79–3·88	0·54	0·38–0·74
Swedish background	Male	683	14669360·3	4·43	4·09–4·79	1·00	reference group	686	14850760·5	4·41	4·08–4·76	1·00	reference group
Third generation	Male	92	2115711·4	4·24	3·42–5·26	0·93	0·74–1·15	93	2234778·7	4·11	3·32–5·09	0·90	0·72–1·11
Second generation - high income	Male	32	824792·1	3·80	2·64–5·49	0·83	0·57–1·17	27	720316·7	3·29	2·20–4·93	0·81	0·54–1·17
Second generation - middle income	Male	91	2215400·9	4·10	3·28–5·12	0·87	0·69–1·08	103	2224164·0	4·50	3·64–5·56	0·99	0·80–1·21
Second generation - low income	Male	12	728913·2	1·82	0·97–3·40	0·35	0·18–0·59	12	726882·3	1·73	0·91–3·29	0·35	0·19–0·59
Swedish background	Female	611	13850073·7	4·58	4·21–4·97	1·00	reference group	612	14049094·5	4·57	4·21–4·96	1·00	reference group
Third generation	Female	83	2005545·9	4·63	3·70–5·81	0·94	0·74–1·18	108	2109844·8	5·24	4·29–6·39	1·18	0·96–1·44
Second generation - high income	Female	40	787369·1	5·62	4·05–7·79	1·15	0·82–1·56	29	681484·5	4·53	3·08–6·65	0·97	0·66–1·39
Second generation - middle income	Female	103	2103162·4	5·17	4·18–6·38	1·13	0·91–1·39	97	2106710·5	5·08	4·07–6·33	1·08	0·87–1·33
Second generation - low income	Female	22	691648·5	3·36	2·08–5·43	0·74	0·47–1·11	22	685842·1	3·59	2·22–5·83	0·76	0·48–1·13
**IV-XI. Other Solid Tumours**													
Swedish background	Both Sexes	1607	28519434·0	5·96	5·66–6·27	1·00	reference group	1614	28899855·0	5·95	5·65–6·26	1·00	reference group
Third generation	Both Sexes	216	4121257·3	5·73	4·98–6·60	0·94	0·81–1·08	231	4344623·5	5·58	4·87–6·40	0·96	0·83–1·10
Second generation - high income	Both Sexes	80	1612161·2	5·33	4·22–6·72	0·88	0·70–1·10	76	1401801·2	5·45	4·29–6·93	0·97	0·76–1·21
Second generation - middle income	Both Sexes	228	4318563·3	5·55	4·81–6·42	0·95	0·83–1·09	227	4330874·5	5·70	4·93–6·58	0·96	0·83–1·10
Second generation - low income	Both Sexes	69	1420561·7	5·61	4·30–7·33	0·88	0·69–1·12	78	1412724·4	6·02	4·67–7·75	1·02	0·80–1·27
Swedish background	Male	823	14669360·3	6·03	5·61–6·48	1·00	reference group	813	14850760·5	5·92	5·51–6·36	1·00	reference group
Third generation	Male	99	2115711·4	5·33	4·33–6·56	0·84	0·68–1·03	107	2234778·7	5·26	4·31–6·43	0·88	0·72–1·07
Second generation - high income	Male	37	824792·1	4·85	3·43–6·85	0·80	0·57–1·10	40	720316·7	5·78	4·15–8·04	1·01	0·72–1·37
Second generation - middle income	Male	103	2215400·9	5·00	4·04–6·20	0·85	0·69–1·04	109	2224164·0	5·33	4·32–6·57	0·92	0·75–1·13

abbreviations: ICCC3 - International Classification of Childhood Cancer, third edition, ASR - age-standardized incidence rate, IRR - Incidence rate ratio, CI - Confidence interval; IRR for both sexes calculated from multivariable poisson regression adjusted for sex and year of birth. For sex-specific analyses, models were adjusted for year of birth alone.

Supplementary Table 5. Cancer incidence between ages 1 and 19 in the years 1991 through 2021 in Sweden by World Bank income level of maternal and paternal country of birth

		Father						Mother					
Migrant Background	Sex	Cases	Person-years	ASR per 100,000 persons	95% CI	IRR	95% CI	Cases	Person-years	ASR per 100,000 persons	95% CI	IRR	95% CI
Second generation - low income	Male	45	728913·2	7·25	5·21–10·08	1·14	0·83–1·53	45	726882·3	7·15	5·14–9·95	1·18	0·86–1·57
Swedish background	Female	784	13850073·7	5·88	5·46–6·33	1·00	reference group	801	14049094·5	5·97	5·55–6·42	1·00	reference group
Third generation	Female	117	2005545·9	6·16	5·09–7·46	1·03	0·85–1·25	124	2109844·8	5·92	4·91–7·14	1·03	0·85–1·24
Second generation - high income	Female	43	787369·1	5·83	4·25–8·00	0·97	0·70–1·29	36	681484·5	5·11	3·61–7·23	0·93	0·65–1·27
Second generation - middle income	Female	125	2103162·4	6·13	5·05–7·45	1·06	0·87–1·27	118	2106710·5	6·08	4·98–7·43	0·99	0·81–1·20
Second generation - low income	Female	24	691648·5	3·89	2·47–6·12	0·62	0·40–0·91	33	685842·1	4·81	3·25–7·14	0·86	0·59–1·19

abbreviations: ICCC3 - International Classification of Childhood Cancer, third edition, ASR - age-standardized incidence rate, IRR - Incidence rate ratio, CI - Confidence interval; IRR for both sexes calculated from multivariable poisson regression adjusted for sex and year of birth. For sex-specific analyses, models were adjusted for year of birth alone.

Supplementary Table 6. Cancer subtype incidence between ages 1 and 19 in the years 1991 through 2021 in Sweden by migrant background

Migrant Background	Cases	Person-years	ASR per 100,000 persons	95% CI	IRR	95% CI
**Acute lymphoid leukaemias**						
Swedish background	932	23615680	3·30	3·09–3·53	1·00	reference group
Third generation	245	6678479	3·10	2·72–3·54	0·92	0·80–1·06
Second generation	218	4852453	3·65	3·16–4·23	1·06	0·91–1·23
2·5 generation	212	4714534	3·66	3·18–4·21	1·10	0·95–1·28
**Acute myeloid leukaemias**						
Swedish background	142	23615680	0·57	0·48–0·68	1·00	reference group
Third generation	48	6678479	0·61	0·46–0·83	1·20	0·86–1·65
Second generation	42	4852453	0·86	0·62–1·20	1·47	1·02–2·05
2·5 generation	30	4714534	0·66	0·45–0·96	1·07	0·71–1·56
**Chronic myeloid leukaemia, MDS, and unspecified leukaemias**						
Swedish background	105	23615680	0·46	0·37–0·56	1·00	reference group
Third generation	22	6678479	0·31	0·20–0·48	0·74	0·46–1·15
Second generation	9	4852453	0·21	0·10–0·44	0·43	0·20–0·81
2·5 generation	22	4714534	0·53	0·34–0·82	1·07	0·66–1·66
**Hodgkin Lymphoma**						
Swedish background	192	23615680	1·07	0·93–1·24	1·00	reference group
Third generation	64	6678479	1·29	1·00–1·65	1·20	0·90–1·58
Second generation	57	4852453	1·86	1·42–2·43	1·64	1·21–2·19
2·5 generation	41	4714534	1·31	0·96–1·79	1·14	0·80–1·57
**Non-hodgkin lymphoma**						
Swedish background	134	23615680	0·60	0·50–0·71	1·00	reference group
Third generation	44	6678479	0·76	0·56–1·03	1·17	0·82–1·63
Second generation	28	4852453	0·71	0·47–1·06	1·09	0·71–1·61
2·5 generation	24	4714534	0·52	0·34–0·79	0·93	0·59–1·40
**Burkitt and misc lymphoma**						
Swedish background	109	23615680	0·48	0·40–0·58	1·00	reference group
Third generation	28	6678479	0·43	0·29–0·64	0·88	0·58–1·29
Second generation	21	4852453	0·41	0·26–0·65	0·91	0·57–1·39
2·5 generation	18	4714534	0·40	0·24–0·66	0·77	0·46–1·22
**Ependymomas**						
Swedish background	87	23615680	0·32	0·26–0·40	1·00	reference group

abbreviations: ICCC3 - International Classification of Childhood Cancer, third edition, ASR - age-standardized incidence rate, IRR - Incidence rate ratio, CI - Confidence interval,CNS - Central nervous system, MDS - myelodysplastic syndrome; IRR calculated from multivariable poisson regression adjusted for sex and year of birth.

Supplementary Table 6. Cancer subtype incidence between ages 1 and 19 in the years 1991 through 2021 in Sweden by migrant background

Migrant Background	Cases	Person-years	ASR per 100,000 persons	95% CI	IRR	95% CI
Third generation	29	6678479	0·43	0·29–0·63	1·17	0·76–1·77
Second generation	12	4852453	0·21	0·11–0·40	0·65	0·34–1·15
2·5 generation	22	4714534	0·41	0·26–0·63	1·25	0·76–1·96
**Astrocytomas and other gliomas**						
Swedish background	415	23615680	1·72	1·55–1·90	1·00	reference group
Third generation	118	6678479	1·68	1·39–2·03	1·00	0·82–1·23
Second generation	73	4852453	1·54	1·19–1·97	0·85	0·66–1·09
2·5 generation	64	4714534	1·32	1·02–1·71	0·77	0·59–0·99
**Intracranial and intraspinal embryonal**						
Swedish background	194	23615680	0·71	0·62–0·83	1·00	reference group
Third generation	43	6678479	0·53	0·39–0·73	0·78	0·55–1·07
Second generation	33	4852453	0·54	0·38–0·78	0·79	0·54–1·13
2·5 generation	38	4714534	0·72	0·52–1·01	0·96	0·67–1·34
**Other and unspecified CNS**						
Swedish background	378	23615680	1·76	1·59–1·96	1·00	reference group
Third generation	110	6678479	1·90	1·56–2·30	1·03	0·83–1·27
Second generation	79	4852453	1·93	1·51–2·46	1·05	0·82–1·34
2·5 generation	68	4714534	1·77	1·37–2·27	0·92	0·70–1·18

abbreviations: ICCC3 - International Classification of Childhood Cancer, third edition, ASR - age-standardized incidence rate, IRR - Incidence rate ratio, CI - Confidence interval,CNS - Central nervous system, MDS - myelodysplastic syndrome; IRR calculated from multivariable poisson regression adjusted for sex and year of birth.

Supplementary Table 7. Cancer subtype incidence between ages 1 and 19 in the years 1991 through 2021 in Sweden by by WHO income level of maternal and paternal country of birth

	Father						Mother					
Migrant Background	Cases	Person-years	ASR per 100,000 persons	95% CI	IRR	95% CI	Cases	Person-years	ASR per 100,000 persons	95% CI	IRR	95% CI
**Acute lymphoid leukaemias**												
Swedish background	1124	28519434·0	3·30	3·10–3·50	1·00	reference group	1138	28899855·0	3·29	3·10–3·50	1·00	reference group
Third generation	172	4121257·3	3·45	2·95–4·04	1·04	0·88–1·22	152	4344623·5	2·99	2·53–3·54	0·87	0·73–1·03
2nd gen - WHO European Region	145	3185678·7	3·88	3·27–4·62	1·12	0·94–1·33	156	3050937·3	4·14	3·51–4·89	1·26	1·07–1·49
2nd gen - WHO Region of the Americas	22	571378·7	3·30	2·14–5·10	0·94	0·60–1·40	29	506788·3	4·54	3·12–6·61	1·40	0·94–1·98
2nd gen - WHO Eastern Mediterranean Region	94	2004243·8	3·56	2·86–4·44	1·10	0·88–1·35	83	1711796·3	3·73	2·93–4·75	1·13	0·89–1·40
2nd gen - WHO African Region	14	595054·9	2·32	1·32–4·07	0·55	0·31–0·90	16	495179·3	3·09	1·81–5·25	0·74	0·44–1·18
2nd gen - WHO Western Pacific Region	13	260674·9	3·67	2·07–6·50	1·19	0·66–1·97	22	449972·4	4·08	2·60–6·40	1·17	0·74–1·73
2nd gen - WHO South-East Asia Region	11	192061·0	4·25	2·24–8·06	1·31	0·68–2·25	19	402914·0	3·57	2·21–5·77	1·08	0·66–1·65
**Acute myeloid leukaemias**												
Swedish background	174	28519434·0	0·57	0·49–0·67	1·00	reference group	182	28899855·0	0·60	0·52–0·70	1·00	reference group
Third generation	30	4121257·3	0·65	0·45–0·95	1·20	0·80–1·74	29	4344623·5	0·56	0·38–0·82	1·06	0·70–1·55
2nd gen - WHO European Region	24	3185678·7	0·79	0·51–1·21	1·24	0·79–1·86	23	3050937·3	0·74	0·47–1·14	1·20	0·76–1·82
2nd gen - WHO Region of the Americas	5	571378·7	0·94	0·35–2·51	1·45	0·51–3·16	<5	506788·3	0·71	0·21–2·38	0·95	0·23–2·49
2nd gen - WHO Eastern Mediterranean Region	14	2004243·8	0·73	0·41–1·27	1·17	0·64–1·94	14	1711796·3	0·81	0·46–1·44	1·33	0·73–2·21
2nd gen - WHO African Region	5	595054·9	0·97	0·39–2·40	1·40	0·50–3·07	5	495179·3	0·87	0·33–2·29	1·64	0·58–3·59
2nd gen - WHO Western Pacific Region	<5	260674·9	1·30	0·41–4·07	1·91	0·47–5·02	6	449972·4	1·55	0·67–3·59	2·15	0·85–4·44
2nd gen - WHO South-East Asia Region	<5	192061·0	0·28	0·04–1·97	0·87	0·05–3·89	<5	402914·0	0·35	0·08–1·42	0·81	0·13–2·53
**Chronic myeloid leukaemia, MDS, and unspecified leukaemias**												
Swedish background	126	28519434·0	0·45	0·38–0·54	1·00	reference group	120	28899855·0	0·42	0·35–0·50	1·00	reference group
Third generation	15	4121257·3	0·37	0·21–0·63	0·83	0·47–1·37	17	4344623·5	0·42	0·26–0·69	0·95	0·55–1·53
2nd gen - WHO European Region	10	3185678·7	0·36	0·19–0·70	0·72	0·35–1·30	12	3050937·3	0·38	0·21–0·71	0·96	0·50–1·66
2nd gen - WHO Region of the Americas	<5	571378·7	0·21	0·03–1·49	0·40	0·02–1·80	<5	506788·3	0·35	0·05–2·47	0·48	0·03–2·16
2nd gen - WHO Eastern Mediterranean Region	<5	2004243·8	0·16	0·05–0·51	0·35	0·09–0·93	<5	1711796·3	0·19	0·05–0·80	0·29	0·05–0·92

abbreviations: WHO - World Health Organization, ICCC3 - International Classification of Childhood Cancer, third edition, ASR - age-standardized incidence rate, IRR - Incidence rate ratio, CI - Confidence interval; IRR for both sexes calculated from multivariable poisson regression adjusted for sex and year of birth.

Supplementary Table 7. Cancer subtype incidence between ages 1 and 19 in the years 1991 through 2021 in Sweden by by WHO income level of maternal and paternal country of birth

	Father						Mother					
Migrant Background	Cases	Person-years	ASR per 100,000 persons	95% CI	IRR	95% CI	Cases	Person-years	ASR per 100,000 persons	95% CI	IRR	95% CI
2nd gen - WHO African Region	<5	595054·9	0·31	0·07–1·39	0·79	0·13–2·48	<5	495179·3	0·00	NA	0·00	0·00–0·00
2nd gen - WHO Western Pacific Region	<5	260674·9	0·69	0·10–4·90	0·89	0·05–3·98	<5	449972·4	0·73	0·21–2·56	1·65	0·41–4·38
2nd gen - WHO South-East Asia Region	<5	192061·0	0·28	0·04–1·97	1·24	0·07–5·54	<5	402914·0	1·23	0·35–4·28	1·89	0·46–5·00
**Hodgkin Lymphoma**												
Swedish background	226	28519434·0	1·05	0·92–1·20	1·00	reference group	248	28899855·0	1·14	1·00–1·29	1·00	reference group
Third generation	46	4121257·3	1·52	1·13–2·04	1·46	1·05–1·98	34	4344623·5	1·11	0·79–1·56	0·94	0·65–1·32
2nd gen - WHO European Region	35	3185678·7	1·59	1·14–2·23	1·45	1·00–2·04	31	3050937·3	1·41	0·99–2·03	1·23	0·83–1·76
2nd gen - WHO Region of the Americas	7	571378·7	1·89	0·88–4·03	1·65	0·70–3·24	<5	506788·3	1·28	0·48–3·45	0·99	0·30–2·32
2nd gen - WHO Eastern Mediterranean Region	21	2004243·8	1·74	1·12–2·70	1·53	0·95–2·33	25	1711796·3	2·46	1·64–3·70	2·01	1·29–2·97
2nd gen - WHO African Region	6	595054·9	1·47	0·64–3·37	1·46	0·58–3·01	<5	495179·3	0·88	0·26–2·91	0·84	0·21–2·20
2nd gen - WHO Western Pacific Region	<5	260674·9	0·69	0·10–4·90	0·54	0·03–2·37	<5	449972·4	1·41	0·51–3·88	1·17	0·36–2·74
2nd gen - WHO South-East Asia Region	<5	192061·0	3·41	1·10–10·59	2·38	0·59–6·25	<5	402914·0	2·18	0·82–5·80	1·42	0·44–3·34
**Non-hodgkin lymphoma**												
Swedish background	170	28519434·0	0·64	0·55–0·75	1·00	reference group	161	28899855·0	0·58	0·50–0·68	1·00	reference group
Third generation	19	4121257·3	0·46	0·29–0·74	0·79	0·47–1·23	34	4344623·5	0·95	0·67–1·35	1·43	0·97–2·04
2nd gen - WHO European Region	20	3185678·7	0·66	0·42–1·05	1·08	0·66–1·67	15	3050937·3	0·51	0·30–0·87	0·90	0·51–1·48
2nd gen - WHO Region of the Americas	<5	571378·7	0·32	0·07–1·36	0·61	0·10–1·90	<5	506788·3	0·36	0·08–1·54	0·73	0·12–2·29
2nd gen - WHO Eastern Mediterranean Region	14	2004243·8	0·95	0·54–1·67	1·26	0·70–2·10	15	1711796·3	1·31	0·76–2·25	1·70	0·96–2·80
2nd gen - WHO African Region	<5	595054·9	0·55	0·13–2·28	0·61	0·10–1·90	<5	495179·3	0·11	0·02–0·76	0·40	0·02–1·76
2nd gen - WHO Western Pacific Region	<5	260674·9	0·00	NA	0·00	0·00–0·00	<5	449972·4	0·00	NA	0·00	0·00–0·00
2nd gen - WHO South-East Asia Region	<5	192061·0	0·45	0·06–3·16	0·97	0·05–4·30	<5	402914·0	0·62	0·20–1·91	1·47	0·36–3·89
**Burkitt and misc lymphoma**												
Swedish background	127	28519434·0	0·46	0·39–0·55	1·00	reference group	136	28899855·0	0·49	0·41–0·59	1·00	reference group
Third generation	15	4121257·3	0·41	0·24–0·70	0·84	0·49–1·35	19	4344623·5	0·44	0·28–0·71	0·83	0·50–1·30
2nd gen - WHO European Region	18	3185678·7	0·54	0·33–0·88	1·27	0·77–1·97	13	3050937·3	0·42	0·23–0·76	0·86	0·48–1·43

abbreviations: WHO - World Health Organization, ICCC3 - International Classification of Childhood Cancer, third edition, ASR - age-standardized incidence rate, IRR - Incidence rate ratio, CI - Confidence interval; IRR for both sexes calculated from multivariable poisson regression adjusted for sex and year of birth.

Supplementary Table 7. Cancer subtype incidence between ages 1 and 19 in the years 1991 through 2021 in Sweden by by WHO income level of maternal and paternal country of birth

	Father						Mother					
**Migrant Background**	**Cases**	**Person-years**	**ASR per 100,000 persons**	**95% CI**	**IRR**	**95% CI**	**Cases**	**Person-years**	**ASR per 100,000 persons**	**95% CI**	**IRR**	**95% CI**
2nd gen - WHO Region of the Americas	<5	571378·7	0·88	0·32–2·41	1·40	0·43–3·32	<5	506788·3	0·41	0·10–1·66	0·73	0·12–2·29
2nd gen - WHO Eastern Mediterranean Region	5	2004243·8	0·20	0·08–0·49	0·67	0·28–1·33	<5	1711796·3	0·20	0·08–0·54	0·62	0·24–1·28
2nd gen - WHO African Region	6	595054·9	0·98	0·39–2·43	1·95	0·76–4·04	<5	495179·3	0·81	0·23–2·84	1·06	0·26–2·80
2nd gen - WHO Western Pacific Region	<5	260674·9	0·00	NA	0·00	0·00–0·00	<5	449972·4	0·00	NA	0·00	0·00–0·00
2nd gen - WHO South-East Asia Region	<5	192061·0	0·45	0·06–3·16	0·98	0·06–4·37	<5	402914·0	0·35	0·08–1·42	0·86	0·14–2·71
**Ependymomas**												
Swedish background	109	28519434·0	0·34	0·28–0·42	1·00	reference group	111	28899855·0	0·33	0·28–0·40	1·00	reference group
Third generation	18	4121257·3	0·41	0·25–0·67	1·13	0·67–1·82	17	4344623·5	0·41	0·25–0·68	1·01	0·59–1·64
2nd gen - WHO European Region	12	3185678·7	0·33	0·18–0·60	0·97	0·51–1·70	15	3050937·3	0·42	0·24–0·72	1·27	0·71–2·10
2nd gen - WHO Region of the Americas	<5	571378·7	0·11	0·02–0·77	0·45	0·03–2·02	<5	506788·3	0·24	0·03–1·68	0·51	0·03–2·26
2nd gen - WHO Eastern Mediterranean Region	5	2004243·8	0·18	0·07–0·46	0·63	0·22–1·40	5	1711796·3	0·25	0·10–0·65	0·74	0·26–1·63
2nd gen - WHO African Region	<5	595054·9	0·22	0·03–1·56	0·43	0·02–1·91	<5	495179·3	0·00	NA	0·00	0·00–0·00
2nd gen - WHO Western Pacific Region	<5	260674·9	0·34	0·05–2·40	0·98	0·06–4·38	<5	449972·4	0·54	0·11–2·54	1·13	0·19–3·56
2nd gen - WHO South-East Asia Region	<5	192061·0	0·28	0·04–1·97	1·30	0·07–5·84	<5	402914·0	0·14	0·02–1·00	0·62	0·04–2·79
**Astrocytomas and other gliomas**												
Swedish background	507	28519434·0	1·72	1·57–1·89	1·00	reference group	491	28899855·0	1·66	1·52–1·82	1·00	reference group
Third generation	60	4121257·3	1·41	1·08–1·83	0·82	0·62–1·06	86	4344623·5	1·86	1·49–2·32	1·16	0·92–1·45
2nd gen - WHO European Region	54	3185678·7	1·59	1·20–2·11	0·95	0·71–1·25	49	3050937·3	1·50	1·11–2·01	0·94	0·69–1·25
2nd gen - WHO Region of the Americas	6	571378·7	0·85	0·37–1·99	0·59	0·23–1·20	6	506788·3	1·01	0·44–2·33	0·69	0·27–1·42
2nd gen - WHO Eastern Mediterranean Region	30	2004243·8	1·60	1·09–2·35	0·83	0·56–1·18	26	1711796·3	1·50	0·99–2·27	0·89	0·58–1·29
2nd gen - WHO African Region	9	595054·9	1·69	0·84–3·40	0·84	0·40–1·53	6	495179·3	1·38	0·60–3·17	0·71	0·28–1·44
2nd gen - WHO Western Pacific Region	<5	260674·9	0·82	0·20–3·33	0·43	0·07–1·33	9	449972·4	1·91	0·95–3·84	1·17	0·56–2·13
2nd gen - WHO South-East Asia Region	<5	192061·0	1·14	0·16–8·08	0·29	0·02–1·28	<5	402914·0	1·14	0·37–3·55	0·58	0·18–1·35
**Intracranial and intraspinal embryonal**												
Swedish background	225	28519434·0	0·68	0·59–0·77	1·00	reference group	231	28899855·0	0·70	0·61–0·79	1·00	reference group

abbreviations: WHO - World Health Organization, ICCC3 - International Classification of Childhood Cancer, third edition, ASR - age-standardized incidence rate, IRR - Incidence rate ratio, CI - Confidence interval; IRR for both sexes calculated from multivariable poisson regression adjusted for sex and year of birth.

Supplementary Table 7. Cancer subtype incidence between ages 1 and 19 in the years 1991 through 2021 in Sweden by by WHO income level of maternal and paternal country of birth

	Father						Mother					
Migrant Background	Cases	Person-years	ASR per 100,000 persons	95% CI	IRR	95% CI	Cases	Person-years	ASR per 100,000 persons	95% CI	IRR	95% CI
Third generation	28	4121257·3	0·59	0·40–0·87	0·85	0·56–1·24	31	4344623·5	0·61	0·42–0·88	0·88	0·60–1·26
2nd gen - WHO European Region	29	3185678·7	0·79	0·54–1·15	1·14	0·76–1·64	22	3050937·3	0·62	0·40–0·96	0·89	0·56–1·34
2nd gen - WHO Region of the Americas	5	571378·7	1·14	0·45–2·86	1·09	0·39–2·36	<5	506788·3	0·59	0·16–2·14	0·72	0·18–1·89
2nd gen - WHO Eastern Mediterranean Region	15	2004243·8	0·56	0·33–0·95	0·90	0·51–1·47	14	1711796·3	0·59	0·34–1·02	0·97	0·54–1·60
2nd gen - WHO African Region	<5	595054·9	0·41	0·12–1·40	0·61	0·15–1·60	<5	495179·3	0·32	0·10–1·00	0·72	0·18–1·88
2nd gen - WHO Western Pacific Region	<5	260674·9	0·56	0·14–2·32	0·94	0·16–2·93	<5	449972·4	0·19	0·03–1·37	0·27	0·02–1·19
2nd gen - WHO South-East Asia Region	<5	192061·0	0·28	0·04–1·97	0·62	0·04–2·75	<5	402914·0	0·28	0·07–1·13	0·58	0·10–1·82
**Other and unspecified CNS**												
Swedish background	453	28519434·0	1·75	1·59–1·93	1·00	reference group	465	28899855·0	1·79	1·63–1·97	1·00	reference group
Third generation	69	4121257·3	2·03	1·59–2·60	1·06	0·82–1·36	67	4344623·5	1·77	1·38–2·27	0·97	0·74–1·24
2nd gen - WHO European Region	50	3185678·7	1·75	1·30–2·34	1·00	0·74–1·32	51	3050937·3	1·79	1·33–2·40	1·05	0·78–1·39
2nd gen - WHO Region of the Americas	15	571378·7	3·24	1·89–5·56	1·68	0·96–2·71	9	506788·3	2·22	1·10–4·47	1·13	0·54–2·05
2nd gen - WHO Eastern Mediterranean Region	34	2004243·8	2·08	1·43–3·02	1·11	0·77–1·55	33	1711796·3	2·36	1·60–3·46	1·25	0·86–1·76
2nd gen - WHO African Region	<5	595054·9	0·22	0·03–1·56	0·11	0·01–0·48	<5	495179·3	0·45	0·11–1·86	0·26	0·04–0·82
2nd gen - WHO Western Pacific Region	7	260674·9	3·44	1·52–7·79	1·74	0·74–3·39	8	449972·4	2·42	1·14–5·16	1·14	0·52–2·14
2nd gen - WHO South-East Asia Region	<5	192061·0	1·69	0·51–5·61	1·03	0·26–2·70	7	402914·0	2·27	1·00–5·15	1·14	0·49–2·22

abbreviations: WHO - World Health Organization, ICCC3 - International Classification of Childhood Cancer, third edition, ASR - age-standardized incidence rate, IRR - Incidence rate ratio, CI - Confidence interval; IRR for both sexes calculated from multivariable poisson regression adjusted for sex and year of birth.

Supplementary Table 8. Cancer subtype incidence between ages 1 and 19 in the years 1991 through 2021 in Sweden by World Bank income level of maternal and paternal country of birth

	Father						Mother					
**Migrant Background**	**Cases**	**Person-years**	**ASR per 100,000 persons**	**95% CI**	**IRR**	**95% CI**	**Cases**	**Person-years**	**ASR per 100,000 persons**	**95% CI**	**IRR**	**95% CI**
**Acute lymphoid leukaemias**												
Swedish background	1124	28519434	3·30	3·10–3·50	1·00	reference group	1138	28899855	3·29	3·10–3·50	1·00	reference group
Third generation	172	4121257	3·45	2·95–4·04	1·04	0·88–1·22	152	4344624	2·99	2·53–3·54	0·87	0·73–1·03
Second generation - high income	77	1612161	4·03	3·19–5·09	1·21	0·95–1·51	81	1401801	4·63	3·68–5·81	1·48	1·17–1·84
Second generation - middle income	193	4318563	3·65	3·13–4·25	1·06	0·91–1·24	216	4330875	4·03	3·48–4·66	1·18	1·02–1·36
Second generation - low income	42	1420562	2·46	1·77–3·42	0·69	0·50–0·92	43	1412724	2·43	1·75–3·36	0·70	0·51–0·94
**Acute myeloid leukaemias**												
Swedish background	174	28519434	0·57	0·49–0·67	1·00	reference group	182	28899855	0·60	0·52–0·70	1·00	reference group
Third generation	30	4121257	0·65	0·45–0·95	1·20	0·80–1·74	29	4344624	0·56	0·38–0·82	1·07	0·71–1·55
Second generation - high income	11	1612161	0·82	0·44–1·54	1·12	0·57–1·96	8	1401801	0·57	0·27–1·20	0·90	0·41–1·71
Second generation - middle income	33	4318563	0·73	0·50–1·05	1·27	0·86–1·83	32	4330875	0·75	0·51–1·09	1·20	0·81–1·72
Second generation - low income	14	1420562	1·01	0·57–1·79	1·65	0·91–2·75	17	1412724	1·11	0·66–1·88	1·97	1·15–3·14
**Chronic myeloid leukaemia, MDS, and unspecified leukaemias**												
Swedish background	126	28519434	0·45	0·38–0·54	1·00	reference group	120	28899855	0·42	0·35–0·50	1·00	reference group
Third generation	15	4121257	0·37	0·21–0·63	0·83	0·47–1·37	17	4344624	0·42	0·26–0·69	0·95	0·55–1·53
Second generation - high income	8	1612161	0·60	0·30–1·23	1·13	0·51–2·15	10	1401801	0·68	0·35–1·32	1·71	0·84–3·09
Second generation - middle income	8	4318563	0·18	0·09–0·40	0·43	0·19–0·83	11	4330875	0·32	0·16–0·62	0·63	0·32–1·12
Second generation - low income	<5	1420562	0·18	0·04–0·90	0·33	0·05–1·04	<5	1412724	0·15	0·02–1·04	0·18	0·01–0·79
**Hodgkin Lymphoma**												
Swedish background	226	28519434	1·05	0·92–1·20	1·00	reference group	248	28899855	1·14	1·00–1·29	1·00	reference group
Third generation	46	4121257	1·52	1·13–2·04	1·46	1·05–1·98	34	4344624	1·11	0·79–1·56	0·94	0·65–1·33
Second generation - high income	15	1612161	1·37	0·82–2·29	1·17	0·67–1·91	12	1401801	1·14	0·64–2·04	0·97	0·51–1·65
Second generation - middle income	53	4318563	1·88	1·42–2·48	1·74	1·28–2·34	51	4330875	1·84	1·38–2·44	1·57	1·15–2·11
Second generation - low income	12	1420562	1·48	0·83–2·65	1·26	0·67–2·16	11	1412724	1·43	0·78–2·62	1·09	0·56–1·89
**Non-hodgkin lymphoma**												
Swedish background	170	28519434	0·64	0·55–0·75	1·00	reference group	161	28899855	0·58	0·50–0·68	1·00	reference group
Third generation	19	4121257	0·46	0·29–0·74	0·79	0·47–1·23	34	4344624	0·95	0·67–1·35	1·43	0·97–2·04

abbreviations: ICCC3 - International Classification of Childhood Cancer, third edition, ASR - age-standardized incidence rate, IRR - Incidence rate ratio, CI - Confidence interval; IRR for both sexes calculated from multivariable poisson regression adjusted for sex and year of birth. For sex-specific analyses, models were adjusted for year of birth alone.

Supplementary Table 8. Cancer subtype incidence between ages 1 and 19 in the years 1991 through 2021 in Sweden by World Bank income level of maternal and paternal country of birth

	Father						Mother					
**Migrant Background**	**Cases**	**Person-years**	**ASR per 100,000 persons**	**95% CI**	**IRR**	**95% CI**	**Cases**	**Person-years**	**ASR per 100,000 persons**	**95% CI**	**IRR**	**95% CI**
Second generation - high income	12	1612161	0·80	0·44–1·45	1·25	0·66–2·15	6	1401801	0·40	0·17–0·92	0·76	0·30–1·57
Second generation - middle income	27	4318563	0·77	0·51–1·15	1·12	0·73–1·65	25	4330875	0·76	0·50–1·16	1·11	0·71–1·66
Second generation - low income	<5	1420562	0·23	0·08–0·66	0·51	0·16–1·22	7	1412724	0·36	0·17–0·77	0·97	0·41–1·92
**Burkitt and misc lymphoma**												
Swedish background	127	28519434	0·46	0·39–0·55	1·00	reference group	136	28899855	0·49	0·41–0·59	1·00	reference group
Third generation	15	4121257	0·41	0·24–0·70	0·84	0·49–1·35	19	4344624	0·44	0·28–0·71	0·83	0·50–1·30
Second generation - high income	8	1612161	0·54	0·26–1·11	1·15	0·54–2·13	<5	1401801	0·35	0·13–0·97	0·55	0·17–1·31
Second generation - middle income	25	4318563	0·53	0·35–0·81	1·27	0·83–1·87	20	4330875	0·42	0·26–0·68	0·95	0·60–1·45
Second generation - low income	<5	1420562	0·15	0·04–0·63	0·27	0·04–0·85	<5	1412724	0·09	0·01–0·67	0·12	0·01–0·55
**Ependymomas**												
Swedish background	109	28519434	0·34	0·28–0·42	1·00	reference group	111	28899855	0·33	0·28–0·40	1·00	reference group
Third generation	18	4121257	0·41	0·25–0·67	1·13	0·67–1·82	17	4344624	0·41	0·25–0·68	1·01	0·59–1·64
Second generation - high income	7	1612161	0·36	0·17–0·78	1·13	0·48–2·26	8	1401801	0·47	0·22–0·97	1·49	0·67–2·86
Second generation - middle income	11	4318563	0·18	0·10–0·34	0·65	0·33–1·15	12	4330875	0·24	0·13–0·44	0·70	0·37–1·23
Second generation - low income	<5	1420562	0·34	0·11–0·99	0·71	0·22–1·69	<5	1412724	0·34	0·12–1·01	0·71	0·22–1·70
**Astrocytomas and other gliomas**												
Swedish background	507	28519434	1·72	1·57–1·89	1·00	reference group	491	28899855	1·66	1·52–1·82	1·00	reference group
Third generation	60	4121257	1·41	1·08–1·83	0·82	0·62–1·06	86	4344624	1·86	1·49–2·32	1·16	0·92–1·46
Second generation - high income	25	1612161	1·49	0·99–2·25	0·87	0·57–1·27	19	1401801	1·36	0·84–2·19	0·80	0·49–1·22
Second generation - middle income	70	4318563	1·63	1·26–2·10	0·91	0·70–1·16	75	4330875	1·65	1·30–2·11	1·02	0·79–1·29
Second generation - low income	11	1420562	0·94	0·49–1·81	0·43	0·22–0·75	10	1412724	0·90	0·45–1·79	0·42	0·21–0·73
**Intracranial and intraspinal embryonal**												
Swedish background	225	28519434	0·68	0·59–0·77	1·00	reference group	231	28899855	0·70	0·61–0·79	1·00	reference group
Third generation	28	4121257	0·59	0·40–0·87	0·85	0·56–1·24	31	4344624	0·61	0·42–0·88	0·88	0·60–1·26
Second generation - high income	13	1612161	0·69	0·39–1·22	1·02	0·55–1·71	9	1401801	0·55	0·28–1·07	0·81	0·38–1·48
Second generation - middle income	37	4318563	0·77	0·55–1·09	1·04	0·72–1·46	33	4330875	0·64	0·44–0·92	0·91	0·62–1·30
Second generation - low income	6	1420562	0·26	0·11–0·58	0·51	0·20–1·04	6	1412724	0·23	0·10–0·51	0·50	0·20–1·04

abbreviations: ICCC3 - International Classification of Childhood Cancer, third edition, ASR - age-standardized incidence rate, IRR - Incidence rate ratio, CI - Confidence interval; IRR for both sexes calculated from multivariable poisson regression adjusted for sex and year of birth. For sex-specific analyses, models were adjusted for year of birth alone.

Supplementary Table 8. Cancer subtype incidence between ages 1 and 19 in the years 1991 through 2021 in Sweden by World Bank income level of maternal and paternal country of birth

	Father						Mother					
**Migrant Background**	**Cases**	**Person-years**	**ASR per 100,000 persons**	**95% CI**	**IRR**	**95% CI**	**Cases**	**Person-years**	**ASR per 100,000 persons**	**95% CI**	**IRR**	**95% CI**
**Other and unspecified CNS**												
Swedish background	453	28519434	1·75	1·59–1·93	1·00	reference group	465	28899855	1·79	1·63–1·97	1·00	reference group
Third generation	69	4121257	2·03	1·59–2·60	1·06	0·82–1·36	67	4344624	1·77	1·38–2·27	0·97	0·74–1·24
Second generation - high income	27	1612161	2·15	1·45–3·18	1·06	0·70–1·52	20	1401801	1·52	0·95–2·43	0·88	0·54–1·34
Second generation - middle income	76	4318563	2·03	1·59–2·60	1·15	0·89–1·45	80	4330875	2·25	1·77–2·86	1·19	0·93–1·51
Second generation - low income	13	1420562	1·03	0·56–1·91	0·60	0·33–1·00	14	1412724	1·16	0·64–2·12	0·65	0·36–1·06

abbreviations: ICCC3 - International Classification of Childhood Cancer, third edition, ASR - age-standardized incidence rate, IRR - Incidence rate ratio, CI - Confidence interval; IRR for both sexes calculated from multivariable poisson regression adjusted for sex and year of birth. For sex-specific analyses, models were adjusted for year of birth alone.
